# Plant jasmonate ZIM domain genes: shedding light on structure and expression patterns of *JAZ* gene family in sugarcane

**DOI:** 10.1186/s12864-017-4142-3

**Published:** 2017-10-11

**Authors:** Feng Liu, Tingting Sun, Ling Wang, Weihua Su, Shiwu Gao, Yachun Su, Liping Xu, Youxiong Que

**Affiliations:** 1 Key Laboratory of Sugarcane Biology and Genetic Breeding, Ministry of Agriculture, Fujian Agriculture and Forestry University, Fuzhou, 350002 China; 2 Key Laboratory of Ministry of Education for Genetics, Breeding and Multiple Utilization of Crops, College of Crop Science, Fujian Agriculture and Forestry University, Fuzhou, 350002 China

**Keywords:** *ScJAZ*, Sugarcane-*Sporisorium scitamineum* interaction, Adversity stimuli, Expression profiles, Antimicrobial action

## Abstract

**Background:**

Sugarcane smut caused by *Sporisorium scitamineum* is one of the most severe fungal diseases in the sugarcane industry. Using a molecular biological technique to mine sugarcane resistance genes can provide gene resources for further genetic engineering of sugarcane disease-resistant breeding. Jasmonate ZIM (zinc-finger inflorescence meristem) domain (JAZ) proteins, which involved in the responses to plant pathogens and abiotic stresses, are important signaling molecules of the jasmonic acid (JA) pathway.

**Results:**

Seven differentially expressed sugarcane *JAZ* genes, *ScJAZ1–ScJAZ7*, were mined from the transcriptome of sugarcane after inoculation with *S. scitamineum*. Bioinformatic analyses revealed that these seven *ScJAZ* genes encoded basic proteins that contain the TIFY and CCT_2 domains. Quantitative reverse transcription polymerase chain reaction (qRT-PCR) analysis demonstrated that the *ScJAZ1–ScJAZ7* genes were tissue specific and differentially expressed under adverse stress. During *S. scitamineum* infection, the transcripts of *ScJAZ4* and *ScJAZ5* were both upregulated in the susceptible genotype ROC22 and the resistant genotype Yacheng05–179; *ScJAZ1*, *ScJAZ2*, *ScJAZ3*, and *ScJAZ7* were downregulated in Yacheng05–179 and upregulated in ROC22; and the expression of *ScJAZ6* did not change in ROC22, but was upregulated in Yacheng05–179. The transcripts of the seven *ScJAZ* genes were increased by the stimuli of salicylic acid and abscisic acid, particularly methyl jasmonate. The expression of the genes *ScJAZ1–ScJAZ7* was immediately upregulated by the stressors hydrogen peroxide, sodium chloride, and copper chloride, whereas slightly induced after treatment with calcium chloride and polyethylene glycol. In addition, the expression of *ScJAZ6*, as well as seven tobacco immunity-associated marker genes were upregulated, and antimicrobial activity against *Pseudomonas solanacearum* and *Fusarium solani* var. *coeruleum* was observed during the transient overexpression of *ScJAZ6* in *Nicotiana benthamiana*, suggesting that the *ScJAZ*6 gene is associated with plant immunity.

**Conclusions:**

The different expression profiles of the *ScJAZ1–ScJAZ7* genes during *S. scitamineum* infection, the positive response of *ScJAZ1–ScJAZ7* to hormones and abiotic treatments, and the function analysis of the *ScJAZ6* gene revealed their involvement in the defense against biotic and abiotic stresses. The findings of the present study facilitate further research on the *ScJAZ* gene family especially their regulatory mechanism in sugarcane.

**Electronic supplementary material:**

The online version of this article (10.1186/s12864-017-4142-3) contains supplementary material, which is available to authorized users.

## Background

Sugarcane (*Saccharum* spp.), one of the most important sugar crops in the world, is also an energy crop for bioenergy production [1]. Sugarcane smut, which is caused by *Sporisorium scitamineum*, has become one of the most severe fungal diseases in sugarcane [2]. Smut disease induces earlier germination of buds, thinner stalks, and higher incidence of tiller and black whip, which decrease cane yield and sugar quality [3, 4]. In Queensland, Australia, smut disease has resulted in severe yield loss and even total crop failure despite suitable climatic conditions [5], which include a temperature range of 25 °C–30 °C and humidity of >80% [6]. *Sporisorium scitamineum* quarantine, the development of smut-resistant sugarcane varieties, and integrated field management are some of the proposed methods of controlling smut disease. Cultivating resistant sugarcane varieties is considered as the most effective measure to control smut disease compared to agronomic practices and chemical treatments [3]. However, it takes 10–12 years to obtain a valuable sugarcane cultivar through conventional crossbreeding, which is mainly attributable to its complex genetic background with a polyploid-aneuploid genome [7–9]. Recently, the continuous development of transgenic technology has provided an effective approach to determining the function of target genes and bio-orientation improvement [10]. Therefore, using molecular biological techniques to mine sugarcane resistance genes may provide excellent gene resources for further genetic engineering of sugarcane disease resistance breeding.

Previous studies have mainly focused on sugarcane resistance to smut disease from the aspects of morphology [11], physiology [12], biochemistry [13], cytology [8], genomics [14], and proteomics [15, 16]. At the molecular level, the smut pathogen in sugarcane could be detected by using conventional PCR [17, 18], TaqMan real-time PCR [19], and loop-mediated isothermal amplification (LAMP) [20, 21]. By using next-generation sequencing technology, the genomes of three strains causing sugarcane smut disease have been completed in China (strain 2014001) [14], Brazil (strain SSC39B) [22], and Germany (strain SscI8) [23], thereby providing novel insights into the pathogenic mechanisms underlying sugarcane smut disease. During *S. scitamineum* infection, 136 transcript-derived fragments (TDFs) [24] and 62 differentially expressed genes [25] in sugarcane were identified by cDNA-amplified fragment length polymorphism (cDNA-AFLP). Furthermore, sugarcane smut resistance-related genes such as transcription factors *X1* and thaumatin (*PR5*) gene [26], chitinase family genes [27], β-1,3-glucanase genes [28, 29], and catalase gene [30] have been cloned and identified. Que et al. [31], Barnabas et al. [16], and Su et al. [15] have investigated the interaction between sugarcane and *S. scitamineum* at the proteome level. Two-dimensional electrophoresis (2DE) coupled with matrix-assisted laser desorption ionization/tandem time-of-flight mass spectrometry (MALDI-TOF/TOF-MS) has been utilized in assessing the expression of some proteins in sugarcane after *S. scitamineum* challenge, and a putative effector of *S. scitamineum* has also been detected [16, 31].

The signal molecule jasmonic acid (JA) plays an important role in plant growth, development, secondary metabolism, and environmental stress response [32–36]. Recent studies have suggested that jasmonate ZIM (zinc-finger inflorescence meristem) domain (JAZ) proteins in the JA signaling pathway may be involved in the generation of a response to plant pathogen attacks [37]. Coronatine insensitive 1 (COI1), which plays a vital part in JA synthesis or perception, is identified in *Arabidopsis thaliana* mutants that are insensitive to JA [38–40]. Investigations have revealed that the *COI1* gene encodes an F-box protein, which is a component of the E3-type ubiquitin ligase SCF (Skp/Cullin/F-box) complex [39, 41]. However, the mechanism by which COI1 transduces the JA signal remains unclear. JAZ family have been characterized as SCF^COI1^ substrates [42–44]; however, its members also acts as repressors of JA signals, which directly interact with a JA-responsive transcription factor, the basic helix-loop-helix leucine zipper-type (bHLH zip-type) factor MYC2 [43, 45]. Therefore, the COI1-JAZ-MYC2 is considered as the first core signal module of the JA pathway [46]. JAZ proteins are also involved in other plant hormone pathways such as gibberellic acid, auxin, ethylene, and salicylic acid (SA) [47, 48].

The TIFY family includes four protein subfamilies, namely, ZIM-like (ZML), TIFY, PEAPOD (PPD), and JAZ [49]. JAZ proteins contain a conserved ZIM domain (TIF[F/Y]XG) near its N-terminal and a Jas motif (SLX_2_FX_2_KRX_2_RX_5_PY) at the C-terminal [44, 50]. The Jas motif consists of 26 amino acids and is the hallmark feature of the JAZ family that serves as a protein-protein interaction surface that is required for the repression of MYC2 and COI1 [43, 51]. Twelve members of the *AtJAZ* genes family have been identified in *A. thaliana* and their expression could be induced by JA, thereby suggesting that these play a feedback regulatory role on the activity of transcription factors that are involved in the JA signaling pathway [42, 43, 52]. In addition, the expression of *AtJAZ* genes are regulated by treatments against plant pathogens, sodium chloride (NaCl), and other stress factors [50, 52]. Previous studies have showed that various *JAZ* genes have different functions; for example, in *A. thaliana*, *JAZ* family genes are expressed during insect herbivory and mechanical damage, except for *AtJAZ11* gene [53]. Demianski et al. [52] reported that eight *AtJAZ* genes are upregulated whereas four *AtJAZ* genes are not differentially expressed during *Pseudomonas syringae* infection in *A. thaliana*. On the other hand, the expression of *OsJAZ* genes in *Oryza sativa* are tissue-specific [54]. Zhu et al. [55] showed that the overexpression of *GsJAZ2* in transgenic *Glycine max* enhances salt resistance. Studies have also revealed that the overexpression of *NaJAZd* and *NaJAZh* genes in *Nicotiana attenuata* inhibits bud formation and promotes nicotine synthesis [56–58].

Current research studies on JAZ proteins mainly focus on model plants such as *A. thaliana* [46] and *O. sativa* [54]. Some of the members of the *JAZ* gene family have also been reported in *Zea mays* [59], *Vitis vinifera* [60], *G. max* [55] and *N. attenuata* [56–58]. However, investigations on the *JAZ* genes and their functions in sugarcane have not been conducted to date. In the present study, seven genes belonging to the *ScJAZ* family were mined from the transcriptomes of sugarcane cultivars Yacheng05–179 (smut-resistant) and ROC22 (smut-susceptible) post inoculation with *S. scitamineum* at 24 h, 48 h, and 120 h [61]. Sequence analysis and phylogenetic relationships were performed to analyze the gene structure and the classification of these *ScJAZ* genes. Their gene expression patterns in different sugarcane tissues, in smut disease, and in the presence of various defense signal compounds as stressors were examined by using quantitative reverse transcription polymerase chain reaction (qRT-PCR). Furthermore, the full-length cDNA sequence of sugarcane *ScJAZ*, hereby designated as *ScJAZ6*, was isolated and characterized by subcellular localization, prokaryotic expression in *Escherichia coli* BL21, spot assay, and transient expression in *Nicotiana benthamiana*. The present study aimed to reveal the structure of the sugarcane *ScJAZ* gene as well as its expression characteristics in response to adversity stress, which may be serve as the foundation in identifying other members of the *ScJAZ* gene family.

## Methods

### Plant materials and treatments

For biotic stress, sugarcane smut-resistant and -susceptible cultivars Yacheng05–179 and ROC22, and smut whips were collected from the Key Laboratory of Sugarcane Biology and Genetic Breeding, Ministry of Agriculture (Fuzhou, China). Robust and healthy stems from the two sugarcane cultivars were harvested, and then soaked in water for 24 h. The two-bud setts of Yacheng05–179 and ROC22 were inoculated with 0.5 μL of a suspension containing 5 × 10^6^ smut spores·mL^−1^ in 0.01% (*v*/*v*) Tween-20, whereas the control was inoculated with 0.01% (*v*/*v*) Tween-20 in sterile distilled water instead of spores [28, 62]. After inoculation, each group containing five buds were randomly selected at 0 h, 24 h, 48 h, and 72 h, and then stored at −80 °C until total RNA extraction. Three biological replicates for each group were prepared.

For tissue-specific expression analysis, six healthy 10-month-old ROC22 plants were selected. The tissue samples, which included the root, +1 leaf (the youngest fully expanded leaf with a visible dewlap), bud, stem pith, and stem epidermis, were collected. Three biological replicates of each tissue were prepared. All samples were fixed in liquid nitrogen and stored at −80 °C until total RNA extraction.

For the abiotic treatments, the 4-month-old ROC22 plantlets were grown hydroponically for one week and then treated with eight exogenous treatments by root dipping at 28 °C with 16 h light and 8 h darkness. The simulated environmental stress conditions included plant hormone stresses of 5 mM SA, 100 μM methyl jasmonate (MeJA), and 100 μM abscisic acid (ABA), oxidative stress of 10 μM hydrogen peroxide (H_2_O_2_), hyper-osmotic stresses of 25% polyethylene glycol (PEG) 8000 and 250 mM NaCl, and heavy metal stresses of 250 mM calcium chloride (CaCl_2_) and 250 mM copper chloride (CuCl_2_) [27]. Whole plantlets treated by plant hormones and CaCl_2_ were collected at 0 h, 3 h, 6 h, and 12 h. Other plantlets were collected at 0 h, 6 h, 12 h, and 24 h after initiation of treatment, except for the 250 mM CuCl_2_ treatment, in which collection was performed at 0 h, 24 h, and 48 h after treatment. Three biological replicates were prepared for each treatment and stored at −80 °C until total RNA extraction.

### RNA extraction and first-strand cDNA synthesis

Total RNA was extracted from all the samples using TRIzol® (Invitrogen, Carlsbad, CA, USA) according to the manufacturer’s protocol, and then analyzed by 1% agarose gel electrophoresis. DNase I (Promega, Madison, WI, USA) was used to remove the residual DNA. Then, 1 μg RNA was used in first-strand cDNA synthesis using a 20-mL reaction volume of the Prime-Script™ RT Reagent Kit (Perfect For Real Time) (TaKaRa, Dalian, China).

### Isolation of sugarcane *ScJAZ* family genes

Seven *ScJAZ* unigenes (*ScJAZ1*, Sugarcane_Unigene_BMK.38081; *ScJAZ2*, Sugarcane_Unigene_BMK.48937; *ScJAZ3*, Sugarcane_Unigene_BMK.47693; *ScJAZ4*, Sugarcane_Unigene_BMK.45854; *ScJAZ5*, Sugarcane_Unigene_BMK.46432; *ScJAZ6*, gi34927808; and *ScJAZ7*, gi35024398) (Table 1), which were determined to be differentially expressed in Yacheng05–179 and ROC22 after inoculation with *S. scitamineum* for 24 h, 48 h, and 120 h in a previous RNA-Seq investigation [61], were selected in this study. One of the full-length *ScJAZ* genes, *ScJAZ6* (gi34927808), which was differently expressed in the sugarcane-resistant genotype but remained unchanged in the susceptible one, was cloned (primers are listed in Table 2) from the cDNA extracted from Yacheng05–179 bud tissue by reverse transcription-polymerase chain reaction (RT-PCR). The amplification procedure was as follows: 94 °C for 4 min; followed by 35 cycles of 94 °C for 30 s, 55 °C for 30 s, and 72 °C for 2 min; and a final 72 °C for 10 min. The RT-PCR product of *ScJAZ6* was gel-purified and cloned into a pMD19-T vector (TaKaRa, Dalian, China), and then sequenced (Shenggong, Shanghai, China).

**Table 1:** A list of differentially expressed *ScJAZ* genes in Yacheng05–179 and ROC22 after challenging with *Sporisorium scitamineum* for 24 h, 48 h, and 120 h, respectively

Gene name	Unigene ID	Yacheng05–179			ROC22		
		Log_2_ fold change (T/CK)			Log_2_ fold change (T/CK)		
		24 h	48 h	120 h	24 h	48 h	120 h
*ScJAZ1*	Sugarcane_Unigene_BMK.38081	5.409	6.560	5.281	2.319	2.782	–
*ScJAZ2*	Sugarcane_Unigene_BMK.48937	2.649	4.206	2.775	2.968	2.843	–
*ScJAZ3*	Sugarcane_Unigene_BMK.47693	–	1.661	–	1.921	–	–
*ScJAZ4*	Sugarcane_Unigene_BMK.45854	–	–	–	–	1.160	1.608
*ScJAZ5*	Sugarcane_Unigene_BMK.46432	–	4.745	3.435	–	–	–
*ScJAZ6*	gi34927808	–	1.845	–	–	–	–
*ScJAZ7*	gi35024398	–	–	–	–	3.240	2.860

In the previous transcriptomics study, after correction, the unigenes with a false discovery rate (FDR) < 0.01 and reads per kb per million reads (RPKM) between samples of <2 (fold-change ≥2) were considered as differentially expressed genes [61]. A log_2_ fold-change indicates the fold change of the differentially expressed genes in the transcriptome [61]. T indicates the transcriptome of sugarcane challenged with *S. scitamineum*. CK represents the transcriptome of the mock material. Yacheng05–179, smut-resistant sugarcane genotype; ROC22, smut-susceptible sugarcane genotype

**Table 2:** Primers used in this study

primer	Forward primer (5′-3′)	Reverse primer (5′-3′)	Strategy
*ScJAZ1*-Q	CCTGTTACTACCACTACTAC	ACAAGGCTTTAGATAGAGTT	qRT-PCR analysis
*ScJAZ2*-Q	CTGAAGAAGGTGAACTCA	ATCTGACTACTAAGCAACA	qRT-PCR analysis
*ScJAZ3*-Q	TTTAATTCGGTGGTTTCC	AATACATCTCTACAGTCTCT	qRT-PCR analysis
*ScJAZ4*-Q	CTTCTACGGCGGCAAGAT	CTTGAGCGACACCTTCCT	qRT-PCR analysis
*ScJAZ5*-Q	CGCTCTGATTCCTGTTCG	GTCTCTTCCTATAATCCTCTTCCT	qRT-PCR analysis
*ScJAZ6*-Q	GCTGTTCCTCCCGTAAGT	GTTGTCACCCTTTCCTTTCTTT	qRT-PCR analysis
*ScJAZ7*-Q	CGATTAGCAGTGATTTCAT	ACATCTCTACAGTCCTCT	qRT-PCR analysis
*ScJAZ6*-cDNA	GGACGAGAAGGTGCTGA	GGCGCTAGGGCAAA	Gene cloning
*ScJAZ6*-Subloc	TGCTCTAGAATGGAGAGGGACTTCCTG	CGGGATCCGATCTGTAGTTTCGTACT	Subcellular localization assay
*ScJAZ6*-32a	CGGGATCCATGGAGAGGGACTTCC	CCCAAGCTTTCAGATCTGTAGTTTCGTACTG	Prokaryotic expression
*ScJAZ6*–1301	TGCTCTAGAATGGAGAGGGACTTCCTG	CGGGATCCTCAGATCTGTAGTTTCGTACT	Transient overexpression
*GAPDH*	CACGGCCACTGGAAGCA	TCCTCAGGGTTCCTGATGCC	Reference genes
*NtHSR201*	CAGCAGTCCTTTGGCGTTGTC	GCTCAGTTTAGCCGCAGTTGTG	Transient overexpression
*NtHSR203*	TGGCTCAACGATTACGCA	GCACGAAACCTGGATGG	Transient overexpression
*NtHSR515*	TTGGGCAGAATAGATGGGTA	TTTGGTGAAAGTCTTGGCTC	Transient overexpression
*NtNPR1*	GGCGAGGAGTCCGTTCTTTAA	TCAACCAGGAATGCCACAGC	Transient overexpression
*NtPR*-1a/c	AACCTTTGACCTGGGACGAC	GCACATCCAACACGAACCGA	Transient overexpression
*NtPR2*	TGATGCCCTTTTGGATTCTATG	AGTTCCTGCCCCGCTTT	Transient overexpression
*NtPR3*	CAGGAGGGTATTGCTTTGTTAGG	CGTGGGAAGATGGCTTGTTGTC	Transient overexpression
*NtEFE26*	CGGACGCTGGTGGCATAAT	CAACAAGAGCTGGTGCTGGATA	Transient overexpression
*NtAccdeaminase*	TCTGAGGTTACTGATTTGGATTGG	TGGACATGGTGGATAGTTGCT	Transient overexpression
*NtEF1*-α	TGCTGCTGTAACAAGATGGATGC	GAGATGGGGACAAAGGGGATT	Transient overexpression

### Sequence analysis of *ScJAZ* family genes

The sequences of the seven *ScJAZ* family genes were translated and analyzed by open reading frame (ORF) Finder (https://www.ncbi.nlm.nih.gov/orffinder/). Their signal peptides and the isoelectric points (pIs) were predicted by using ExPASy (http://us.expasy.org/tools). The conserved domain architecture of the ScJAZ proteins were predicted by using the NCBI conserved domains program (http://www.ncbi.nlm.nih.gov/Structure/cdd/wrpsb.cgi), the SMART program (http://smart.embl-heidelberg.de/), and MOTIF search (http://www.genome.jp/tools/motif/). Psort software (http://psort.hgc.jp/form.html) was used to predict the subcellular location of the ScJAZ6 proteins. ClustalW was employed for multiple sequence alignment of the sugarcane ScJAZ proteins with 12 *A. thaliana* [46], 23 *Z. mays* [59], 15 *O. sativa* [54] and 11 *V. vinifera* [60] homologous proteins, respectively. A phylogenetic tree was constructed by the maximum-likelihood method (1000 bootstrap replicates) using the MEGA 6.06 program [63].

### qRT-PCR analysis

To analyze the expression patterns of the *ScJAZ* family genes in different sugarcane tissues and in response to various adverse stressors, qRT-PCR was conducted using SYBR Green Master (ROX) (Roche, China) and an ABI 7500 qRT-PCR system (Applied Biosystems, South San Francisco, CA, USA). The glyceraldehyde-3-phosphate dehydrogenase (*GAPDH*) gene (primers listed in Table 2) was employed as internal control [64, 65]. Beacon Designer 8.12 software was used to design the specific qRT-PCR primers (Table 2) based on the unigene sequences of *ScJAZ1*–*ScJAZ7*. Each 20-μL qRT-PCR reaction system consisted of the following: 10 μL of the SYBR Green Master Mix, 0.8 μL of each 10 μM forward and reverse primers, 1.0 μL of the cDNA template (20× diluted cDNA), and 7.4 μL of sterile distilled water. The qRT-PCR reaction conditions were as follows: 50 °C for 2 min, 95 °C for 10 min, 40 cycles of 95 °C for 15 s, and 60 °C for 1 min. Each qRT-PCR was repeated three times. The 2^-ΔΔCt^ method was used to analyze the qRT-PCR data [66]. Statistical analysis was conducted using the Data Processing System (DPS) v7.05 software (China). Data were expressed as the mean ± standard error (SE). Significance (*P*-value <0.05) was calculated using one-way ANOVA, followed by Duncan’s new multiple range test.

### Subcellular localization assay

The ORF of *ScJAZ6* without a stop codon (primers are listed in Table 2) was inserted into the two restriction sites (*Xba*I and *Bam*HI) of the pCAMBIA 2300-*GFP* vector. Then, the recombinant vector pCAMBIA 2300*-ScJAZ6*-*GFP* was transformed into the competent cells of *Agrobacterium tumefaciens* strain EHA105. *Agrobacterium*-mediated transient expression in *N. benthamiana* leaf was performed according to Su et al. [67]. Subcellular localization of the fusion protein was observed by using a confocal laser scanning microscope (Leica TCS SP5, Germany) after infiltration for 48 h.

### Prokaryotic expression in *E. coli* BL21 (DE3) cells

The ORF of *ScJAZ6* was amplified by using the ScJAZ6-32a primers (Table 2). The PCR product and the pET 32a (+) vector that were both digested with *Bam*HI and *Hin*dIII were ligated. The recombinant plasmid (pET 32a–*ScJAZ6*) was obtained and then transformed into *E. coli* BL21 (DE3) competent cells. The prokaryotic expression product was gathered after the BL21 pET 32a–*ScJAZ6* cells were induced by 1.0 mM isopropyl β-D-thiogalactoside (IPTG) at 28 °C for 0 h, 2 h, 4 h, 6 h, and 8 h. Moreover, the LB medium with *E. coli* BL21 (blank) and BL21 pET 32a (control) cells were separately induced by IPTG for 0 h and 8 h, and then analyzed by sodium dodecyl sulfate polyacrylamide gel electrophoresis (SDS-PAGE) [67, 68].

As reported, the expression of *JAZ* gene could be regulated by various environmental stimuli such as MeJA [44], salt and alkali stresses [55], PEG [54, 60], and pathogen and pest attacks [69, 70]. To study the expression of *ScJAZ6* in *E. coli* in response to different abiotic conditions, a spot assay was conducted in combination with treatment using PEG 8000, NaCl, and CuCl_2_. The *E. coli* BL21 cells containing pET 32a–*ScJAZ6* or pET 32a (control) were cultured in LB medium at 37 °C until these reached OD_600_ of 0.6. Then, IPTG was added to the cultures at a concentration of 1.0 mM and further cultured at 37 °C for another 12 h. The cultured cells were diluted to an OD_600_ of 0.6 and then again respectively diluted to 10^−3^- and 10^−4^-fold by using the LB medium [68]. Ten microliters from each of the 10^−3^- and 10^−4^-fold dilutions was spotted onto LB plates containing the compound of PEG 8000 (15%, 30%, and 45%), NaCl (250 mM, 500 mM, and 750 mM), or CuCl_2_ (250 mM, 500 mM, and 750 mM) [28]. All plates were cultured at 37 °C overnight and then photographed [68].

### The role of the *ScJAZ6* gene in response to pathogen infection

To study the function of the *ScJAZ6* gene in response to pathogen infection, the overexpression vector pCAMBIA 1301-*ScJAZ6* was constructed (the primers used are listed in Table 2). The recombinant vector and the control were separately transformed into *Agrobacterium* strain EHA105 cells, and then cultured in LB liquid medium (supplemented with 50 μg/mL kanamycin and 35 μg/mL rifampicin) overnight at 28 °C. After incubation, the cells were collected and then re-suspended in MS liquid medium (containing 200 μM acetosyringone) to an OD_600_ of 0.8. The cells were infiltrated into the eight-leaf-old *N. benthamiana* leaves and then cultured at 24 °C for 24 h to 48 h (16 h light/8 h darkness) [67]. The treated *N. benthamiana* leaves were used in ion conductivity measurements, transcripts analysis of the target gene and the nine tobacco immunity-associated marker genes, and DAB (3,3′-diaminobenzidinesolution) staining according to Su et al. [29].

To analyze the inhibitory effect of *ScJAZ6* to pathogens, two important tobacco pathogens, *Pseudomonas solanacearum* and *Fusarium solani* var. *coeruleum*, were cultured to an OD_600_ of 0.8 in potato dextrose water (PDW) liquid medium at 28 °C. Then, the two pathogenic bacteria were separately infected into the *N. benthamiana* leaves that were agroinfiltrated with pCAMBIA 1301-*ScJAZ6* or pCAMBIA 1301 for 24 h. All treatment materials were cultured at 24 °C (16 h light/8 h darkness) for 7 d and then photographed. Each test was repeated three times.

## Results

### Expression profile of *ScJAZ* family genes in sugarcane after inoculation with *S. scitamineum*

In our previous transcriptomics study, the sugarcane smut-resistant genotype Yacheng05–179 and -susceptible genotype ROC22 were challenged with *S. scitamineum* for 24 h, 48 h, and 120 h [61]. Table 1 shows that five *ScJAZ* genes (*ScJAZ1*, *ScJAZ2*, *ScJAZ3*, *ScJAZ5*, and *ScJAZ6*) were upregulated by *S. scitamineum* in the resistant genotype, whereas in the susceptible genotype, five *ScJAZ* genes (*ScJAZ1*, *ScJAZ2*, *ScJAZ3*, *ScJAZ4*, and *ScJAZ7*) were upregulated (Table 1). These findings indicate that the *ScJAZ* genes could be induced by *S. scitamineum* in sugarcane, and their expression profiles in smut-resistant and smut-susceptible sugarcane cultivars after *S. scitamineum* inoculation were different.

### Phylogenetic analysis of sugarcane ScJAZ family

**Fig. 1:**
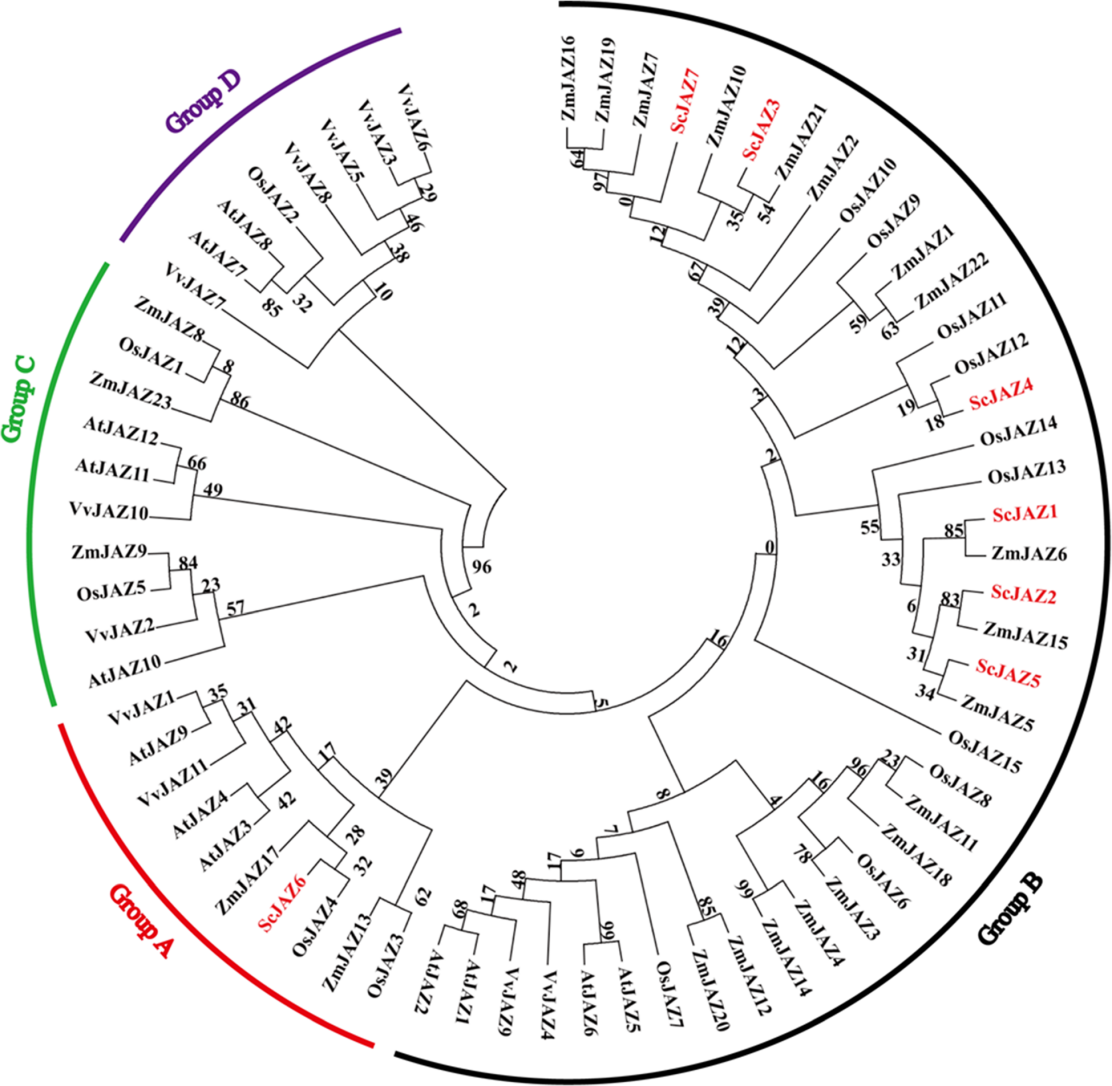
Phylogenetic relationships between sugarcane ScJAZ proteins, and the JAZ family proteins from *Arabidopsis thaliana*, *Zea mays*, *Oryza sativa*, and *Vitis vinifera*. The maximum-likelihood method with 1000 bootstrap replications was used. GenBank Accession Numbers were as follows: AtJAZ1 (NP_564075.1); AtJAZ2 (NP_565096.1); AtJAZ3 (NP_566590.1); AtJAZ4 (NP_175283.2); AtJAZ5 (NP_564019.1); AtJAZ6 (NP_565043.1); AtJAZ7 (NP_181007.1); AtJAZ8 (NP_564349.1); AtJAZ9 (NP_177227.5); AtJAZ10 (NP_568287.1); AtJAZ11 (NP_189930.1); AtJAZ12 (NP_189930.1); ZmJAZ1 (GRMZM2G343157); ZmJAZ2 (GRMZM2G445634); ZmJAZ3 (GRMZM2G117513); ZmJAZ4 (GRMZM2G024680); ZmJAZ5 (GRMZM2G145412); ZmJAZ6 (GRMZM2G145458); ZmJAZ7 (GRMZM2G382794); ZmJAZ8 (GRMZM2G086920); ZmJAZ9 (GRMZM2G145407); ZmJAZ10 (GRMZM2G171830); ZmJAZ11 (GRMZM2G005954); ZmJAZ12 (GRMZM2G101769); ZmJAZ13 (GRMZM2G151519); ZmJAZ14 (GRMZM2G064775); ZmJAZ15 (GRMZM2G173596); ZmJAZ16 (GRMZM2G338829); ZmJAZ17 (GRMZM2G126507); ZmJAZ18 (GRMZM2G116614); ZmJAZ19 (GRMZM2G066020); ZmJAZ20 (GRMZM2G089736); ZmJAZ21 (GRMZM2G036351); ZmJAZ22 (GRMZM2G036288); ZmJAZ23 (GRMZM2G143402); VvJAZ1 (XM_002284819); VvJAZ2 (XM_002262714); VvJAZ3 (XM_003634778); VvJAZ4 (XM_002272327); VvJAZ5 (XM_002277733); VvJAZ6 (XM_002277769); VvJAZ7 (XM_002277916); VvJAZ8 (CBI30922); VvJAZ9 (XM_002277121); VvJAZ10 (XM_002263220); VvJAZ11 (XM_002282652); OsJAZ1 (BAG92077.1); OsJAZ2 (ABF94310.1); OsJAZ3 (ABF94311.1); OsJAZ4 (ABF96426.1); OsJAZ5 (ABF96480.1); OsJAZ6 (BAG98147.1); OsJAZ7 (Q8H395.1); OsJAZ8 (Q69P94.1); OsJAZ9 (Q8GSI0.1); OsJAZ10 (Q10QW3.1); OsJAZ11 (Q8GRS2.1); OsJAZ12 (Q7XEZ1.1); OsJAZ13 (Q7XEZ6.1); OsJAZ14 (Q7XEZ4.1); and OsJAZ15 (Q94LG1.1). The red triangle represents seven sugarcane ScJAZ proteins

The results of phylogenetic analysis of ScJAZ proteins are shown in Fig. 1. Based on the reports of Chini [46] and Zhou et al. [59], the JAZ proteins from sugarcane, *A. thaliana*, *Z. mays*, *O. sativa*, and *V. vinifera* were clustered into four branches (groups A–D). The seven sugarcane ScJAZs were clustered into two branches, group A (ScJAZ6) and group B (ScJAZ1, ScJAZ2, ScJAZ3, ScJAZ4, ScJAZ5 and ScJAZ7).

### Sequence analysis of the sugarcane ScJAZ family

Multiple alignment analysis showed that the seven ScJAZ proteins shared only 23.63% identity at the amino acid sequence level. The domain architecture, pI, and amino acids of these ScJAZ proteins were predicted and shown in Additional file 1: Figure S1. The protein length of ScJAZ1–ScJAZ7 varied from 136 aa to 403 aa. The ScJAZ6 protein was 403 aa in length, which was the longest compared to the other six ScJAZ proteins. The pI features of ScJAZ1–ScJAZ7 were all >7, thereby indicating that these seven ScJAZ proteins are basic proteins. No signal peptide and transmembrane region were observed in the ScJAZ1–ScJAZ7 proteins. Multiple alignment identified two highly conserved sequence motifs in all seven ScJAZ proteins. The TIF[F/Y]XG motif was located at the N-terminal of ScJAZ, which is also known as the ZIM or TIFY domain [50]. Another Jas motif SLX_2_FX_2_KRX_2_RX_5_PY was observed at the C-terminal of the ScJAZ protein, which has been reported to play an important role in regulating jasmonate responses in *A. thaliana* [46].

### Tissue-specific expression patterns of *ScJAZ* family genes

**Fig. 2:**
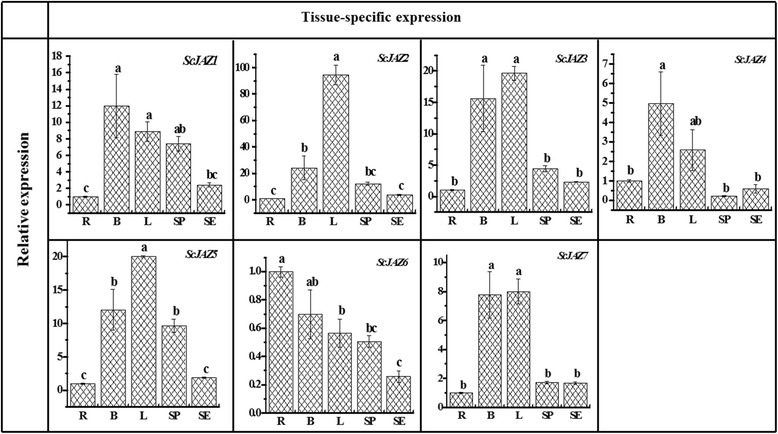
Tissue-specific expression analysis of seven *ScJAZ* family genes in different 10-month-old ROC22 tissues by qRT-PCR. Data was normalized to the *GAPDH* expression level. All data points are the means ± SE (*n* = 3). Different lowercase letters indicate a significant difference, as determined by the Duncan’s new multiple range test (*p*-value <0.05). R: Root; L: Leaf; B: Bud; SP: Stem pith; SE: Stem epidermis

To study the expression patterns of the *ScJAZ* family genes in different tissues, the sugarcane root, bud, leaf, stem pith, and stem epidermis were used. Figure 2 shows that these seven *ScJAZ* genes were successfully detected in all of the five sugarcane tissues. *ScJAZ3*, *ScJAZ4*, and *ScJAZ7* presented a similar gene expression pattern, and the highest expression levels were observed in the bud and leaf, whereas exhibited low expression levels in other three tissues (root, stem pith, and stem epidermis). *ScJAZ1* showed the highest expression levels in the bud, leaf, and stem pith, but accumulated at relatively low levels in the root and stem epidermis. A low expression level of *ScJAZ2* and *ScJAZ5* was found to accumulate in the root and stem epidermis, whereas a high level of gene expression was detected in the leaf. Unlike the other six *ScJAZ* genes, *ScJAZ*6 was expressed at low level in sugarcane tissues, and the highest *ScJAZ*6 transcript levels were detected in the root and bud than the other tissues. These findings suggest that these seven *ScJAZ* genes were constitutively expressed in all five sugarcane tissues, and most of them were abundant in the bud and leaf tissues.

### Transcripts of *ScJAZ* family genes in sugarcane post inoculation with *S. scitamineum*

**Fig. 3:**
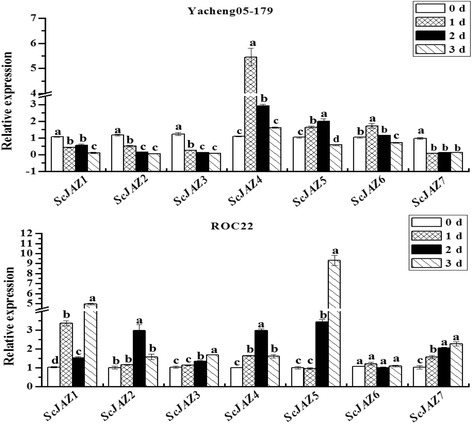
Expression analysis of seven sugarcane *ScJAZ* family genes during sugarcane-*Sporisorium scitamineum* interaction by qRT-PCR. Data was normalized to the *GAPDH* expression level. All data points (with the deduction of their mocks) were expressed as the mean ± SE (*n* = 3). Different lowercase letters indicate a significant difference, as determined by the Duncan’s new multiple range test (*p*-value <0.05). Yacheng05–179, smut-resistant sugarcane genotype; ROC22, smut-susceptible sugarcane genotype

The expression patterns of the *ScJAZ* family genes in response to *S. scitamineum* stress are shown in Fig. 3. The qRT-PCR results suggest that in Yacheng05–179, with elongated treatment time, the RNA expression of the four *ScJAZ* genes (*ScJAZ*1, *ScJAZ2*, *ScJAZ3*, and *ScJAZ7*) was downregulated after infection with *S. scitamineum*, whereas the other two genes (*ScJAZ4* and *ScJAZ6*) were significantly upregulated and reached the highest expression at 1 d. Furthermore, the expression of the remaining gene, *ScJAZ5*, peaked at 2 d and then decreased at 3 d. However, in ROC22, the six *ScJAZ* genes (*ScJAZ1*, *ScJAZ2*, *ScJAZ3*, *ScJAZ4*, *ScJAZ5*, and *ScJAZ7*) were upregulated after inoculation, and only *ScJAZ6* showed no change in transcript abundance after inoculation. The expression of *ScJAZ1* and *ScJAZ7* rapidly increased at 1 d after inoculation, which then peaked at 3 d. The expression of *ScJAZ3* and *ScJAZ5* showed no change after inoculation 1 d, but maintained at relatively high levels at 3 d. The expression levels of *ScJAZ2* and *ScJAZ4* increased and peaked at 2 d after inoculation. Comparison of the expression of the *ScJAZ* genes in the two sugarcane varieties indicated that the transcripts of both *ScJAZ4* and *ScJAZ5* were upregulated in the smut-susceptible (ROC22) and smut-resistant (Yacheng05–179) genotypes after infection throughout the whole process, whereas that of *ScJAZ1*, *ScJAZ2*, *ScJAZ3*, and *ScJAZ7* showed opposite expression patterns, and that of *ScJAZ6* showed no significant difference in ROC22 but was upregulated at 1 d and downregulated in Yacheng05–179 at 3 d. These findings suggest that the expression of the *ScJAZ* genes was all induced after infection with *S. scitamineum* regardless of genotype, except for *ScJAZ6* in ROC22, but showed different expression patterns.

### Expression of the *ScJAZ* family genes in response to various defense-related signal compounds

**Fig. 4:**
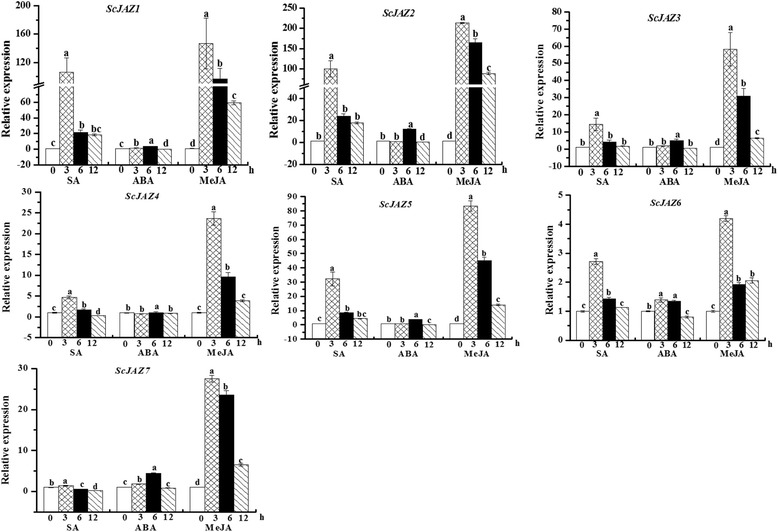
qRT-PCR analysis of seven sugarcane *ScJAZ* family genes in 4-month-old ROC22 plantlets after treatment with 5 mM SA, 100 μM MeJA and 100 μM ABA. Data was normalized to the *GAPDH* expression level. All data points were the means ± SE (*n* = 3). Different lowercase letters indicate a significant difference, as determined by the Duncan’s new multiple range test (*p*-value <0.05). SA, salicylic acid; MeJA, methyl jasmonate; ABA, abscisic acid

Figure 4 shows that the expression patterns of the *ScJAZ* family genes in response to phytohormones such as SA, MeJA, and ABA. The seven members of the *ScJAZ* gene family were expressed after the application of SA, ABA, and MeJA. Under the SA and MeJA stimuli, the *ScJAZ1–ScJAZ7* genes were rapidly upregulated and reached their peak values at 3 h, and were then gradually downregulated from 6 h to 12 h after treatment. Moreover, the transcripts of all these seven *ScJAZ* genes significantly varied during MeJA treatment. Following ABA stress application, although the changes in gene expression levels were not higher than the other two treatments, the transcripts of the *ScJAZ1–ScJAZ7* genes increased from 3 h, peaked at 6 h, and then decreased at 12 h. These results revealed that these *ScJAZ* family genes exhibit the same expression pattern under the individual defense-related signal compounds stresses, which elicited a positive response to SA and ABA, particularly to MeJA.

### Expression of *ScJAZ* family genes in response to various abiotic stressors

**Fig. 5:**
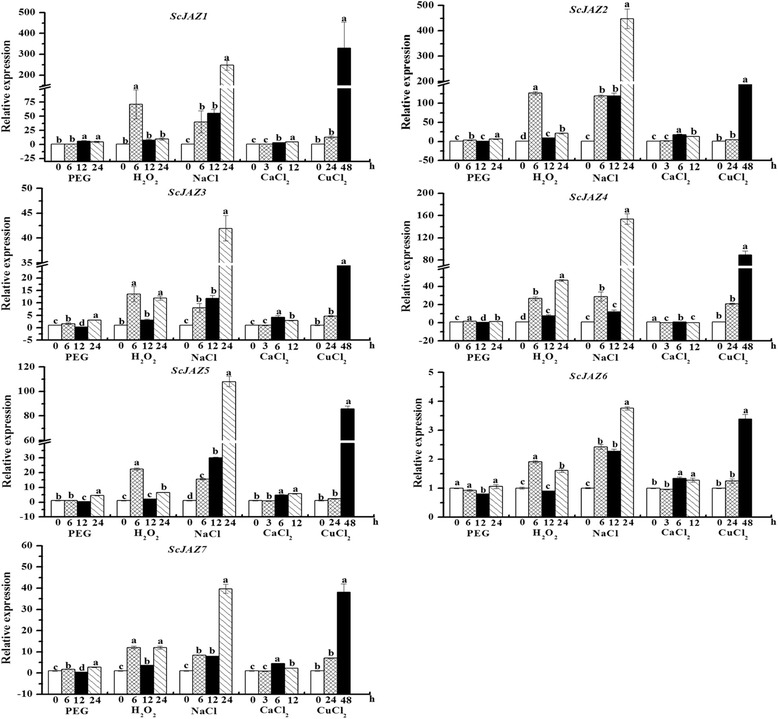
qRT-PCR analysis of seven sugarcane *ScJAZ* family genes in 4-month-old ROC22 plantlets after treatment with 25% PEG, 10 μM H_2_O_2_, 250 mM NaCl, 100 mM CuCl_2_, and 100 mM CaCl_2_. Data were normalized to the expression level of the *GAPDH* gene. All data points were expressed as the mean ± SE (*n* = 3). Different lowercase letters indicate a significant difference, as determined by the Duncan’s new multiple range test (*p*-value <0.05). PEG, polyethylene glycol; H_2_O_2_, hydrogen peroxide; NaCl, sodium chloride; CaCl_2_, calcium chloride; CuCl_2_, copper chloride

In the case of PEG, NaCl, CuCl_2_, H_2_O_2_, and CaCl_2_ stressors, the *ScJAZ* genes exhibited different levels of expression (Fig. 5). In response to PEG, the *ScJAZ* genes showed different expression patterns, six (*ScJAZ1*, *ScJAZ2*, *ScJAZ3*, *ScJAZ5*, *ScJAZ6*, and *ScJA7*) were upregulated at 24 h except for *ScJAZ4* at 12 h after treatment. All seven *ScJAZ* genes were upregulated after H_2_O_2_ treatment, and most of these peaked at 6 h after exposure. Significant changes in the levels of expression of *ScJAZ1–ScJAZ7* were observed upon NaCl treatment and reached peak values at 24 h after treatment. Meanwhile, minimal changes in the transcript levels of *ScJAZ1–ScJAZ7* were observed for the initial 24 h after treatment, which then sharply increased at 48 h, thereby indicating a positive role in response to CuCl_2_ stress. The expression levels of six *ScJAZ* genes (*ScJAZ1*, *ScJAZ2*, *ScJAZ3*, *ScJAZ5*, *ScJAZ6*, and *ScJAZ7*) slightly increased after CaCl_2_ treatment, whereas the expression of *ScJAZ4* decreased. Interestingly, compared to that under CaCl_2_ and PEG stresses, the expression of the seven *ScJAZ* family genes was remarkably upregulated after H_2_O_2_, NaCl, and CuCl_2_ treatment.

### Isolation of the full-length *ScJAZ6* gene from sugarcane

The present study cloned and identified the full-length sequence of the *ScJAZ6* gene (GenBank Accession No. KX352246). The cDNA length of *ScJAZ6* was 1776 bp, which included an intact ORF (1212 bp, from position 202 to 1413) that encoded a 403-aa polypeptide. Its molecular mass and pI were 42.28 kDa and 9.76, respectively. The NCBI search for conserved protein domains showed that ScJAZ6 contained a TIFY domain and a CCT_2 domain, thereby indicating that ScJAZ6 belongs to the TIFY family of JAZ proteins. The TIF[F/Y]XG motif at the N-terminal of the ScJAZ6 protein was located within the TIFY domain. The Jas motif SLX_2_FX_2_KRX_2_RX_5_PY at the C-terminal was situated within the CCT_2 domain. The amino acid sequence of ScJAZ6 showed 93%, 86%, and 86% homology with that of *Sorghum bicolor*, *Setaria italic*, and *Z. mays* JAZ proteins from NCBI, respectively.

### Subcellular localization of ScJAZ6

**Fig. 6:**
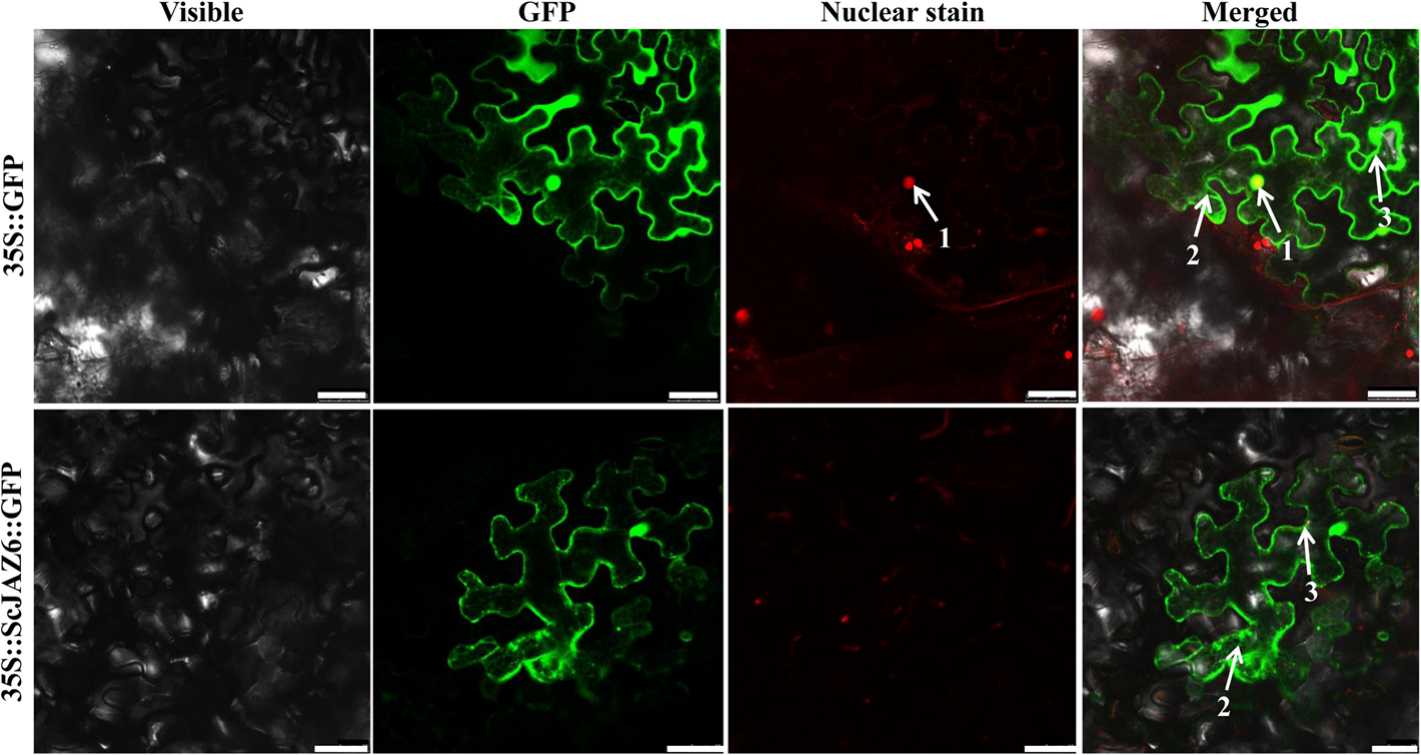
Subcellular localization analysis of ScJAZ6 in *Nicotiana benthamiana* leaves 48 h after infiltration. The epidermal cells were used for taking images of green fluorescence, red fluorescence, visible light, and merged light. Read arrows 1, 2, and 3 indicated nucleus, cytoplasm and plasma membrane, respectively

After *Agrobacterium*-mediated transformation, the subcellular localization of the ScJAZ6 protein in *N. benthamiana* leaves was determined (Fig. 6). After infiltration for 48 h, the fusion protein of ScJAZ6::GFP was located in the cytoplasm and the plasma membrane relative to that in the control.

### *ScJAZ6* expression in *E. coli*

**Fig. 7:**
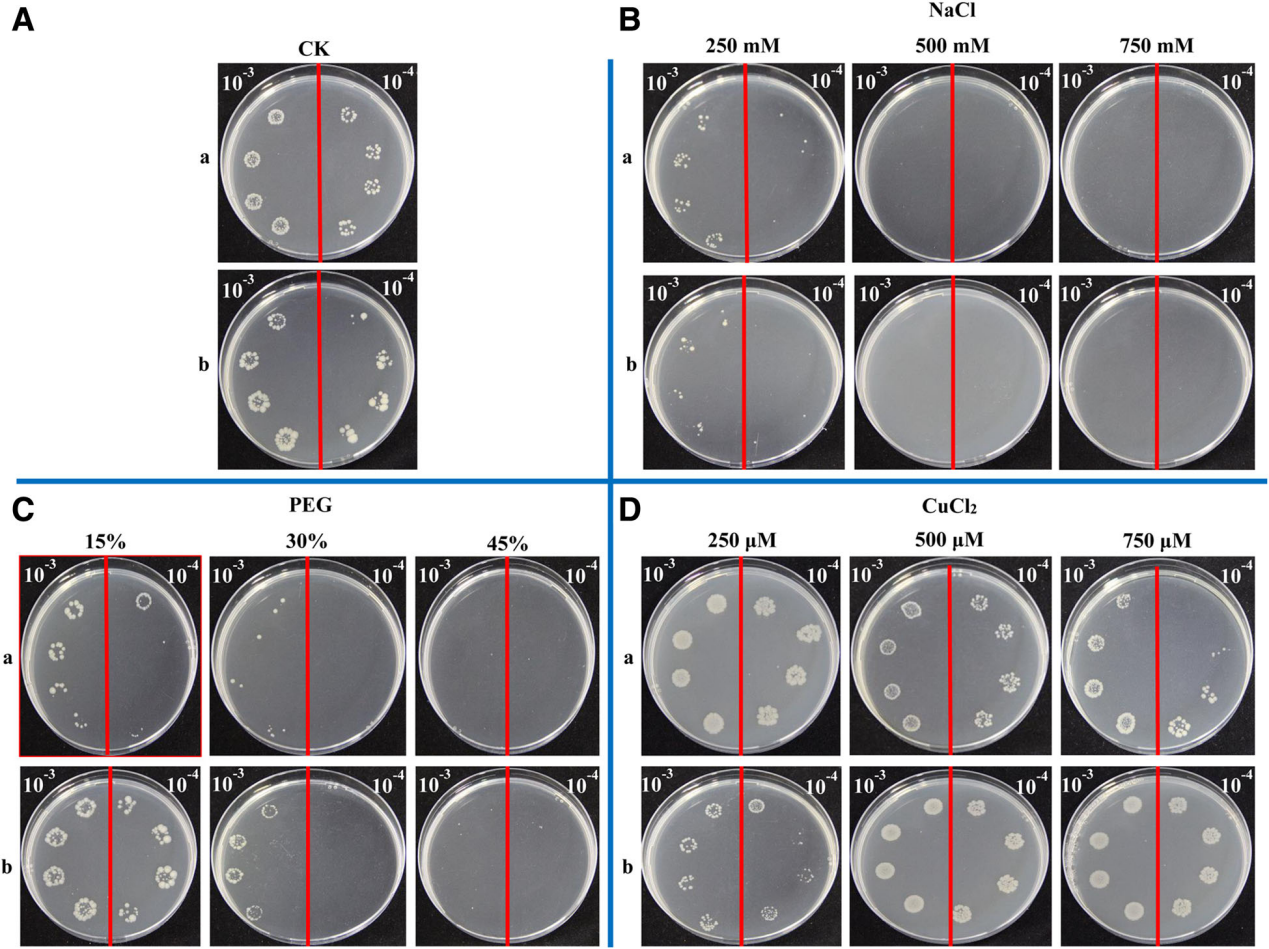
Spot assays of BL21 pET 32a–*ScJAZ6* (**b**) and BL21 pET 32a (control) (**a**) on LB plates supplemented with NaCl, PEG, and CuCl_2_. After induction by using 1.0 mM isopropyl β-D-thiogalactoside (IPTG) at 37 °C for 12 h, the cultures were adjusted to OD_600_ = 0.6. Then 10 μL of the 10^−3^-fold (left side of the red line on the plate) to 10^−4^-fold (right side of the red line on the plate) dilutions were spotted onto the LB plates without any supplement (CK) (**A**) or with NaCl (250 mM, 500 mM, and 750 mM) (**B**), PEG (15%, 30%, and 45%) (**C**) and CuCl_2_ (250 μM, 500 μM, and 750 μM) (**D**), respectively. NaCl, sodium chloride; PEG, polyethylene glycol; CuCl_2_, copper chloride

SDS-PAGE indicated the accumulation of a 50-KDa protein after the BL21 pET 32a–*ScJAZ6* cells were induced by 1 mM IPTG at 28 °C for 2 h, 4 h, 6 h, and 8 h (Additional file 2: Figure S2). The growth of the control cells (BL21 pET 32a) and the gene-expressed cells (BL21 pET 32a–*ScJAZ6*) on LB plates with different supplements was studied (Fig. 7). After 24 h of cultivation, the BL21 pET 32a–*ScJAZ6* cells showed a faster growth rate than that of the control, which was supplemented with PEG and CuCl_2_, but not with NaCl (Fig. 7). Interestingly, the higher concentration of CuCl_2_, the faster the growth of the BL21 pET 32a–*ScJAZ6* cells. The above results indicated that the recombinant protein of ScJAZ6 enhance the tolerance of *E. coli* BL21 cells to PEG, particularly the CuCl_2_ stimulus.

### Transient overexpression of *ScJAZ6* in *N. benthamiana* leaves induces defense response

**Fig. 8:**
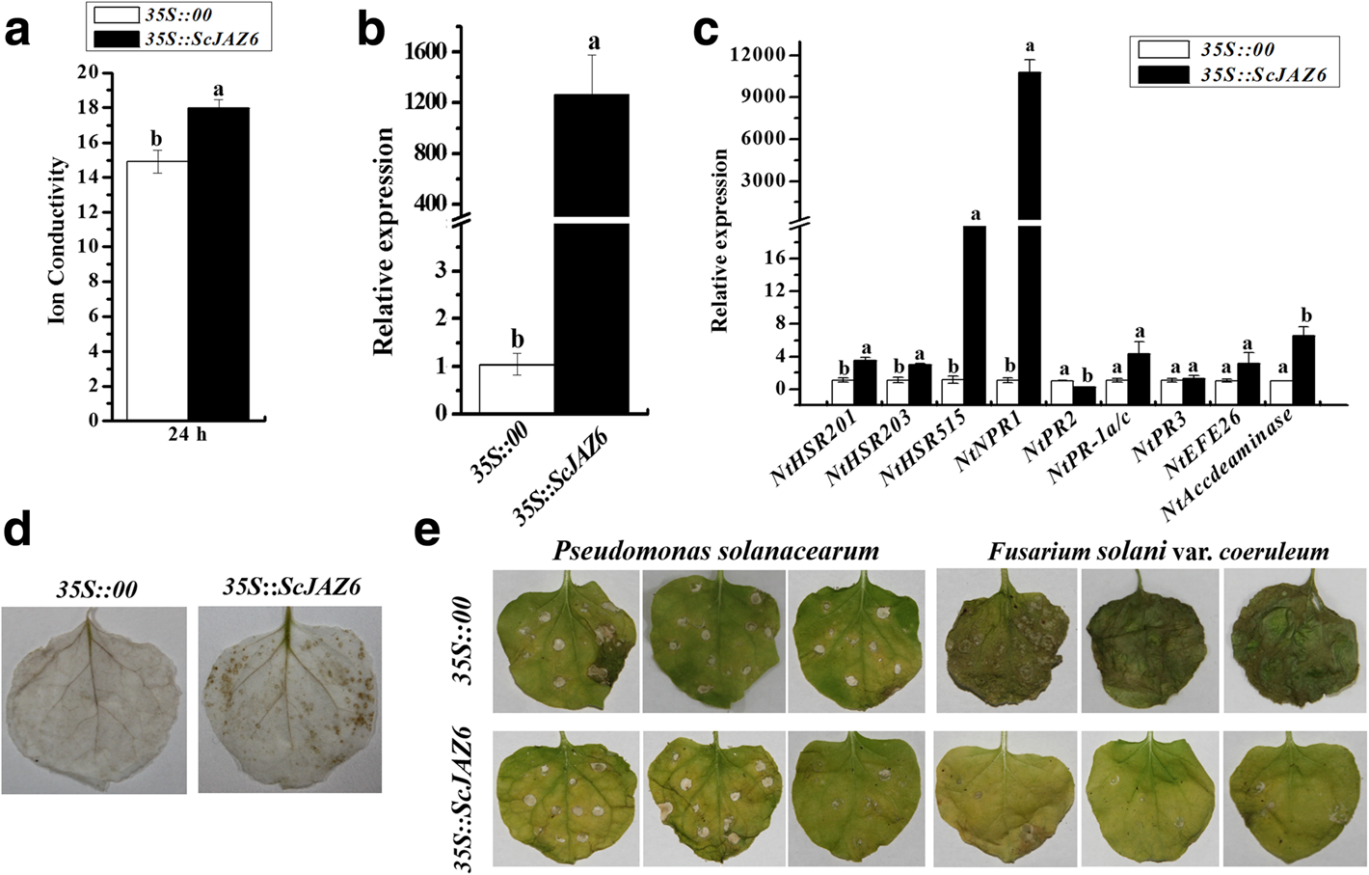
The transient expression of *ScJAZ6* in *Nicotiana benthamiana* leaves. (**a**) Conductivity measurement of *N. Benthamiana* leaves infiltrated with *35S::ScJAZ6*-containing *Agrobacterium* strain for 24 h. (**b** and **c**) The transcripts of *ScJAZ6* and the immunity-associated marker genes in the *N. benthamiana* leaves at 24 h after infiltration. *NtEF1-α* was used for normalization of the transcript levels. *NtHSR201*, *NtHSR203*, and *NtHSR515*, hypersensitive response marker genes; *NtNPR1*, a salicylic acid pathway-related gene; *NtPR2*, *NtPR-1a/c*, and *NtPR3*, jasmonate pathway-associated genes; *NtEFE26* and *NtAccdeaminase*, the ethylene synthesis-dependent genes. Mock, the *Agrobacterium* strain carrying *35S::00*. All data points were expressed as the mean ± SE (*n* = 3). Different lowercase letters indicate a significant difference, as determined by the Duncan’s new multiple range test (p-value <0.05). (**d**) DAB (3,3′-diaminobenzidinesolution) staining of *N. benthamiana* leaves at 48 h after *Agrobacterium* strain infiltration. (**e**) The infection results of *N. benthamiana* leaves by *Pseudomonas solanacearum* and *Fusarium solani* var. *coeruleum* after infiltration with *35S::00* (control) or *35S::ScJAZ6*-containing *Agrobacterium* strain. Disease symptoms of infected leaves were observed at 7 d post-inoculation

The transient overexpression of *ScJAZ6* in *N. benthamiana* leaves was observed (Fig. 8). After infiltration for 1 d, a typical hypersensitive response (HR) was observed in the test leaves compared to that in the control such as enhanced conductivity (Fig. 8a) and darker DAB staining color (Fig. 8d). A significant increase in *ScJAZ6* transcript abundance was observed in the *N. benthamiana* leaves at 24 h after infiltration (Fig. 8b). Moreover, the expression levels of seven tobacco immunity-associated marker genes in *N. benthamiana*, namely, the hypersensitive response (HR) marker genes *NtHSR201*, *NtHSR203* and *NtHSR515*, the SA-related gene *NtNPR1*, *NtPR-1a/c*, and the ethylene synthesis depended genes *NtEFE26* and *NtAccdeaminase*, were upregulated by the transient overexpression of *ScJAZ6* (Fig. 8c).

To further validate the inhibitory effect of *ScJAZ6* to pathogens, *Agrobacterium* EHA105 that harbored *35S::ScJAZ6* was infiltrated into leaves of *N. benthamiana* for 24 h, then two tobacco pathogens, *P. solanacearum* and *F. solani* var. *coeruleum*, were separately injected into *N. benthamiana*. Figure 8e shows that the leaves of the control exhibited more severe disease symptom than that of the *N. benthamiana* leaves with infiltration with *35S::ScJAZ6* after inoculation with *P. solanacearum* or *F. solani* var. *coeruleum* for 7 d. These results suggest that *ScJAZ6* is associated with the HR or immunity of sugarcane, and its overexpression in *N. benthamiana* showed an antimicrobial action on *P. solanacearum* and *F. solani* var. *coeruleum*.

## Discussion

### Isolation of sugarcane *ScJAZ* family genes and phylogenetic analysis

The JAZ subfamily belongs to the TIFY family, which is a plant-specific family of putative transcription factors, plays a significant role in regulating the development and response of plants to abiotic and biotic stresses [49, 50, 71]. To date, JAZ family proteins have been identified and characterized in several plants such as *A. thaliana* [46], *V. vinifera* [60], *O. sativa* [54], *Z. mays* [59], *G. max* [55], and *N. attenuata* [56–58]. However, the expression and function of members of the JAZ family in sugarcane are unknown. The present study aimed to identify *ScJAZ* family genes in sugarcane after *S. scitamineum* inoculation and to systematically analyze their sequence characters and expression profiles under various stress-related conditions.

In the present study, seven *ScJAZ* family genes were identified in sugarcane after *S. scitamineum* inoculation. Multiple alignment analysis showed that all these seven ScJAZ proteins shared a relatively low level of amino acid sequences identity (23.63%), and the number of amino acids varied from 136 to 403. JAZ subfamily proteins with high sequence difference have also been identified in *A. thaliana* [46] and *O. sativa* [54], suggesting that these genes may have undergone major mutations and functional divergence. Sequence analysis showed that all seven ScJAZ proteins contained a conserved ZIM domain and a Jas motif (Additional file 2: Figure S1), which is consistent to the findings of previous reports of Chung et al. [72] and Staswick et al. [73]. Previous studies also demonstrated that several *A. thaliana* JAZ proteins engage in homomeric and heteromeric interactions that are regulated by the TIFY motif (TIFF/YXG) within the ZIM domain [72]. The function of the ZIM domain was to recruit transcriptional co-repressors [74]. Similar to other plant species such as *A. thaliana* [46], *V. vinifera* [60], and *Z. mays* [59], domain architecture analysis showed that all these seven sugarcane ScJAZ proteins contained two conserved domains, namely, the TIFY and CCT_2 domains. The ScJAZ protein family was clustered into two clades (Fig. 1), which were in agreement with the findings in *A. thaliana* [46] and *Z. mays* [59].

### Transcripts of the *ScJAZ* family genes are differentially expressed in various sugarcane tissues

Previous studies have shown that *JAZ* genes are differentially and constitutively expressed in plants such as *Hevea brasiliensis* [75], *Z. mays* [59], and *O. sativa* [54]. Most *HbJAZ* genes (*HbJAZ1*, *HbJAZ2*, *HbJAZ7*, *HbJAZ8*, *HbJAZ9*, *HbJAZ10*, and *HbJAZ11*) in *H. brasiliensis* showed higher levels of expression in the leaves than in the bark [75]. *OsTIFY3* showed constitutively high expression levels in the root, callus, panicle, sheath, and flag leaf [54]. In *Z. mays*, *ZmJAZ13* and *ZmJAZ23* exhibited constitutive expression in the root, stalk, leaf, apex, silk, tassel, and seeds [59]. Similarly, in the present study, the transcripts of the seven *ScJAZ* family genes were differentially expressed in various sugarcane tissues (Fig. 2). *ScJAZ1*, *ScJAZ3*, *ScJAZ4*, and *ScJAZ7* showed the highest expression level in the buds and leaves; *ScJAZ2* and *ScJAZ5* showed the highest levels of expression in the leaves, whereas *ScJAZ6* was highest in roots and buds. These findings indicate that *ScJAZ* family genes are constitutively expressed in sugarcane tissues. Sugarcane buds often serve as the route of entry for smut pathogen infections [29]. Previous studies have shown that when sugarcane is infected with *S. scitamineum*, differential changes in the bud ultrastructure occur, i.e., the cell wall is damaged in susceptible varieties, whereas these remain intact in resistant cultivars [8]. The transcripts of most *ScJAZ* genes were abundant in sugarcane bud and leaf tissues, suggesting that *ScJAZ* genes may take part in the resistance to *S. scitamineum* infections.

### The majority of sugarcane *JAZ* genes shows a positive response to *S. scitamineum* infections

Biotic stressors such as pathogen infection and insect herbivory negatively influences plant growth [3, 4]. Plant defense mechanisms can be mediated by hormone signaling such as JA and SA [76, 77]. Previous studies have shown that the *JAZ* genes are involved in plant pathogen resistance [52]. In *A. thaliana*, the *AtJAZ1–AtJAZ12* genes are induced by *Pseudomonas syringae* infection [52]. Approximately 12 *SlJAZ* genes (*SlJAZ1–SlJAZ12*) in tomato are upregulated by *Pst* DC3000 [37]. In the present study, the transcripts of seven *ScJAZ* genes were induced by *S. scitamineum* infection (except for *ScJAZ6* in ROC22), but showed different expression patterns between the two sugarcane genotypes after smut pathogen inoculation (Fig. 3). In the smut-susceptible genotype (ROC22), the expression of *ScJAZ4*, *ScJAZ5*, and *ScJAZ6* were upregulated by infection, whereas that of *ScJAZ1*, *ScJAZ2*, *ScJAZ3*, and *ScJAZ7* did not changed with infection. In contrast, *ScJAZ1*, *ScJAZ2*, *ScJAZ3*, *ScJAZ4*, *ScJAZ5*, and *ScJAZ7* were downregulated after inoculation, whereas *ScJAZ6* showed no response to *S. scitamineum* infection in the smut-resistant genotype (Yacheng05–179). These findings indicate that the *ScJAZ* genes may be involved in the response to smut pathogen infection and played different roles in the two sugarcane genotypes.

### Sugarcane *JAZ* genes are responsive to various plant hormones and abiotic stresses

A previous study has shown that plant hormones, including SA, JA, and ethylene, act as defense signal compounds for two types of plant-induced resistance, including systemic acquired resistance (SAR) and induced systemic resistance (ISR) [78]. Recent investigations have demonstrated that JA treatment and environmental stress could rapidly trigger the expression of *JAZ* genes, which in turn regulates their responses to JA [42, 43, 79]. In *V. vinifera*, *VvJAZ4*, *VvJAZ5*, and *VvJAZ9* are induced by both JA and MeJA [60]. In the present study, we found that the *ScJAZ1–ScJAZ7* genes were upregulated by SA and MeJA, with the latter showing a more significant induction. *JAZ* genes also play an important role in the JA signaling pathway in *Arabidopsis* and rice [44, 54]. Transgenic *Arabidopsis* overexpresses *JAI3*, which encodes a JAZ protein that harbors a short Jas motif, thereby resulting in an MeJA-insensitive phenotype [44]. Another phytohormone, ABA, also plays an important role in the response of plants to adverse environmental conditions [80]. For instance, four *JAZ* genes reported in *O. sativa* are responsive to ABA [80]. In this study, seven *ScJAZ* genes were all upregulated by ABA at 6 h, thereby suggesting that the expression of these *ScJAZ* genes could be regulated by ABA-dependent signaling pathways [60].

In addition to plant hormones, *JAZ* genes could be induced by various types of abiotic stresses such as wounding [81], herbivores [56], and salt and alkali [55]. In *Z. mays*, the expression of *ZmJAZ14* is induced by salt and PEG [59]. In the present study, all *ScJAZ* genes were immediately induced by H_2_O_2_, NaCl, and CuCl_2_, and slightly induced by CaCl_2_ and PEG (Fig. 4), thereby suggesting that the *ScJAZ* genes may play roles in different types of stress pathways in sugarcane. A recent study has shown that the overexpression of *OsTIFY11a* in rice results in an improvement in stress tolerance such as drought, salt, and low temperature [44].

### The functional verification of *ScJAZ6*: Subcellular localization, prokaryotic expression, and transient overexpression

To date, several plant *JAZ* genes have been characterized in *A. thaliana* [46], *O. sativa* [54], *N. tabacum* [56], and *Glycine soja* [82]. In this study, *ScJAZ6* was upregulated by *S. scitamineum* induction in the resistant genotype Yacheng05–179, but remained unchanged in the susceptible genotype ROC22 (Fig. 3). As reported, the expression of recombinant plant proteins in *E. coli* cells enhances cell growth when under high temperature (47 °C), NaCl, carbofuran, CdCl_2_, CuCl_2_, and UV-B stressors [83]. The expression of the *ScJAZ6* gene resulted in a positive response to the SA, ABA, MeJA, H_2_O_2,_ NaCl and CaCl_2_ stressors (Figs. 4 and 5). Moreover, the transcripts of *ScJAZ6* was also significantly upregulated by CuCl_2_ (Fig. 5), which coincided with the results of the spot assay in that the expression of the recombinant protein of ScJAZ6 in *E. coli* BL21 cells resulted in better growth under CuCl_2_ stress (Fig. 7). Interestingly, the higher concentration of CuCl_2_, the better the cell growth, thereby suggesting that *ScJAZ6* could enhance the tolerance of sugarcane to CuCl_2_. Subcellular localization of ScJAZ6 in *N. benthamiana* showed that 35S::ScJAZ6::GFP was located in the cytoplasm and the plasma membrane (Fig. 6). However, previous studies have shown that *ZmJAZ14* and *GsJAZ2* were only located in the nucleus [55, 59]. Cell death could efficiently restrict pathogen growth and development and trigger some reaction such as the induction of R gene expression, ion fluxes, stimulation of ROS, and defense-related hormones [84–86]. In this study, during transient expression of *ScJAZ6* in *N. benthamiana* leaves, the darker DAB staining color was indicative of the accumulation of H_2_O_2_ in the *N. benthamiana* leaves after 24 h of infiltration (Fig. 8d). As Levine et al. [87] reported, H_2_O_2_ played a key role in evaluating a localized hypersensitive response. Furthermore, the immunity-associated marker genes involved in the HR, SA, JA, and ethylene signaling pathways were upregulated (Figs. 8c and d), which in turn induces a defense response in *N. benthamiana*, and an antimicrobial action against the pathogenic bacteria, namely, *P. solanacearum*, and *F. solani* var. *coeruleum* (Fig. 8e). These results showed that the expression of the *ScJAZ6* gene is closely related to plant immunity and HR, which is in agreement with the findings of Levine et al. [87] and Ron and Avni [88].

## Conclusions

Transcriptome analysis of *S. scitamineum*-resistant and -susceptible sugarcane genotypes respectively challenged with *S. scitamineum* identified seven sugarcane *JAZ* family genes that encoded proteins harboring a N-terminal ZIM domain and C-terminal Jas motif. The *ScJAZ1–ScJAZ7* genes were constitutively expressed in the sugarcane root, bud, leaf, stem pith, and stem epidermis tissues. Transcript expression of these *ScJAZ* genes were observed after infection with *S. scitamineum*, regardless of genotype (except for *ScJAZ6* in ROC22), but were differentially expressed. In the case of phytohormones and various abiotic stresses, the seven *ScJAZ* family genes were all upregulated by the SA, ABA, MeJA, H_2_O_2_, NaCl, and CuCl_2_ stimuli. In addition, the overexpression of *ScJAZ6* in *E. coli* BL21 cells enhanced its growth under CuCl_2_, NaCl, and PEG stimuli. Moreover, the transient overexpression of *ScJAZ6* in *N. benthamiana* leaves resulted in enhanced conductivity, darker DAB staining color, and increased expression levels of the seven tobacco immunity-associated marker genes, as well as an antimicrobial activity. The findings of the present study may be utilized in future research investigations on the function of *JAZ* family genes in sugarcane, and may also serve as a basis for the elucidation of the mechanism underlying sugarcane immunity.

## Additional files

Additional file 1: Figure S1. The domain structure of the corresponding ScJAZ proteins. The conserved motifs of TIFY (TIF[F/Y]XG) and Jas (SLX_2_FX_2_KRX_2_RX_5_PY) presented among the seven sugarcane ScJAZ proteins. Green box: TIFY domain; Blue box: CCT_2 domain. aa: the number of amino acids; pI: isoelectric point. (TIFF 343 kb)

Additional file 2: Figure S2. The prokaryotic expression of pET 32a–*ScJAZ6* fusion protein in *Escherichia coli* BL21 (DE3). M, protein marker; 1, blank (*E. coli* BL21 cells) without induction; 2, blank induction for 8 h; 3, control (BL21 pET 32a) without induction; 4, control induction for 8 h; 5, BL21 pET 32a–*ScJAZ6* without induction; 6–9, BL21 pET 32a–*ScJAZ6* induction for 2 h, 4 h, 6 h, and 8 h, respectively. The induced protein is indicated by a red arrow. (TIFF 3905 kb)

## Abbreviations

2DE: Two-dimensional gradient polyacrylamide gels
aa: Amino acids
ABA: Abscisic acid
bHLH zip-type: Basic helix-loop-helix leucine zipper-type
CaCl_2_: Calcium chloride
cDNA-AFLP: cDNA-amplified fragment length polymorphism
COI1: Coronatine insensitive 1
CuCl_2_: Copper chloride
GAPDH: Glyceraldehyde-3-phosphate dehydrogenase
H_2_O_2_: Hydrogen peroxide
HR: Hypersensitive reaction
IPTG: Isopropyl β-D-thiogalactoside
ISR: Induced systemic resistance
JAZs: Jasmonate ZIM domains
LAMP: Loop-mediated isothermal amplification
MALDI-TOF-TOF/MS: Matrix-assisted laser desorption/ionization time of flight mass spectrometry
MeJA: Methyl jasmonate
NaCl: Sodium chloride
ORF: Open reading frame
pI: isoelectric point
PEG: Polyethylene glycol
PR: Pathogenesis-related protein
qRT-PCR: Quantitative reverse transcription polymerase chain reaction
RNA-seq: RNA sequencing
RT-PCR: Reverse transcription-polymerase chain reaction
SA: Salicylic acid
SAR: Systemic acquired resistance
SCF: Skp/Cullin/F-box
SDS-PAGE: Sodium dodecyl sulfate polyacrylamide gel electrophoresis
SE: Standard error
TDFs: Transcript-derived fragments

## References

[1] Yadav RL, Solomon S (2006). Potential of developing sugarcane by-product based industries in India. Sugar Tech.

[2] Hossain SMI, Eusufzai SUK, Rahman MA. Effect of different irrigation levels on growth and yield parameters of sugarcane. Pak J Agr Res. 2009:28–35.

[3] Sundar AR, Barnabas EL, Malathi P, Viswanathan R, Mworia J (2012). A mini-review on smut disease of sugarcane caused by *Sporisorium scitamineum*. Botany.

[4] Xu LP, Chen RK (2000). Current status and prospects of smut and smut resistance breeding in sugarcane. Fujian J Agr.

[5] Magarey RC, Bull JI, Sheahan T, Denney D, Bruce RC. Yield losses caused by sugarcane smut in several crops in Queensland. Proc Aust Soc Sugar Cane Technol. 2010;32:347–54.

[6] Riley IT, Jubb TF, Egan BT, Croft BJ, Singh V, Kumar V. First outbreak of sugarcane smut in Australia. Proc Int Soc Sugar Cane Technol. 1999;23:333–6.

[7] Liu YQ, Kai CR, Ming GD (1996). Analysis of quantitatine inheritance for smut resiotance in sugarcane. J Fujian Agr Univ.

[8] Solas MT, Pinon D, Vicente C, Legaz ME (1999). Ultrastructural aspects of sugarcane bud infection by *Ustilago scitaminea* teliospores. Sugar Cane.

[9] Scortecci KC, Creste S, Jr TC, Xavier MA, Landell MGA, Figueira A, et al. Challenges, opportunities and recent advances in sugarcane breeding. In: Abdurakhmonov I, editor. Plant Breeding. Rijeka: InTech Publisher; 2012. p. 268–96.

[10] Cui XD, Zhang BY, Ding CJ, Su XH (2013). Plant transgenic technology and its application to genetic improvement of forest trees. World Forestry Res.

[11] Waller JM (1970). Sugarcane smut (*Ustilago scitaminea*) in Kenya : II. Infection and resistance. T Brit Mycol Soc.

[12] Lloyd HL (1983). Chemical assay potentially suitable for determination of smut resistance of sugarcane cultivars. Plant Dis.

[13] Gloria BAD, Albernas MCC, Amorim L (1995). Structural characteristics of buds of sugarcane cultivars with different levels for resistance to smut. Z Pflanzenk Pflanzen.

[14] Que YX, Xu LP, Wu QB, Liu YF, Ling H, Liu YH (2014). Genome sequencing of *Sporisorium scitamineum* provides insights into the pathogenic mechanisms of sugarcane smut. BMC Genomics.

[15] Su YC, Xu LP, Wang ZQ, Peng Q, Yang YT, Yun C, et al. Comparative proteomics reveals that central metabolism changes are associated with resistance against *Sporisorium scitamineum* in sugarcane. BMC Genomics. 2016;17:800. 10.1186/s12864-016-3146-8 PMC5062822 27733120

[16] Barnabas EL, Ashwin N, Kaverinathan K, Trentin AR, Pivato M, Sundar AR (2016). Proteomic analysis of a compatible interaction between sugarcane and *Sporisorium scitamineum*. Proteomics.

[17] Albert HH, Schenck S (1996). PCR amplification from a homolog of the *bE* mating-type gene as a sensitive assay for the presence of *Ustilago scitaminea* DNA. Plant Dis.

[18] Singh N, Somai BM, Pillay D (2004). Smut disease assessment by PCR and microscopy in inoculated tissue cultured sugarcane cultivars. Plant Sci.

[19] Su YC, Wang SS, Guo JL, Xue BT, Xu LP, Que YX. A TaqMan real-time PCR assay for detection and quantification of *Sporisorium scitamineum* in sugarcane. The Scientific World J. 2013;2013:1351–8. 10.1155/2013/942682 PMC3819024 24228020

[20] Su YC, Yang YT, Peng Q, Zhou DG, Chen Y, Wang ZQ, et al. Development and application of a rapid and visual loop-mediated isothermal amplification for the detection of *Sporisorium scitamineum *in sugarcane. Sci Rep. 2016;6:23994. 10.1038/srep23994 PMC4817513 27035751

[21] Shen W, Xu G, Sun L, Zhang L, Jiang Z (2016). Development of a loop-mediated isothermal amplification assay for rapid and sensitive detection of *Sporisorium scitamineum* in sugarcane. Ann Appl Biol.

[22] Taniguti LM, Schaker PD, Benevenuto J, Peters LP, Carvalho G, Palhares A (2015). Complete genome sequence of *Sporisorium scitamineum* and biotrophic interaction transcriptome with sugarcane. PLoS One.

[23] Dutheil JY, Mannhaupt G, Schweizer G, Sieber CM, Münsterkötter M, Güldener U (2016). A tale of genome compartmentalization: the evolution of virulence clusters in smut fungi. Genome Biol Evol.

[24] Que YX, Lin JW, Song XX, Xu LP, Chen RK (2011). Differential gene expression in sugarcane in response to challenge by fungal pathogen *Ustilago scitaminea* revealed by cDNA-AFLP. Biomed Res Int.

[25] Borrás-Hidalgo O, Thomma BPHJ, Carmona E, Borroto CJ, Pujol M, Arencibia A (2005). Identification of sugarcane genes induced in disease-resistant somaclones upon inoculation with *Ustilago scitaminea* or *Bipolaris sacchari*. Plant Physiol Bioch.

[26] Heinze BS, Thokoane LN, Williams NJ, Barnes JM, Rutherford RS (2001). The smut-sugarcane interaction as a model system for the integration of marker discovery and gene isolation. Proc S Afr Sug Technol Ass.

[27] Su YC, Xu LP, Wang SS, Wang ZQ, Yang Y, Chen Y (2015). Identification, phylogeny, and transcript of Chitinase family genes in sugarcane. Sci Rep.

[28] Su YC, Xu LP, Xue BT, Wu QB, Guo JL, Wu LG (2013). Molecular cloning and characterization of two pathogenesis-related β-1,3-glucanase genes *ScGluA1* and *ScGluD1* from sugarcane infected by *Sporisorium scitamineum*. Plant Cell Rep.

[29] Su YC, Wang ZQ, Liu F, Li Z, Peng Q, Guo JL (2016). Isolation and characterization of *ScGluD2*, a new sugarcane beta-1,3-Glucanase D family gene induced by *Sporisorium scitamineum*, ABA, H_2_O_2_, NaCl, and CdCl_2_ stresses. Front Plant Sci.

[30] Su YC, Guo JL, Ling H, Chen SS, Wang SS, Xu LP (2014). Isolation of a novel peroxisomal catalase gene from sugarcane, which is responsive to biotic and abiotic stresses. PLoS One.

[31] Que YX, Xu LP, Lin JW, Ruan MH, Zhang MQ, Chen RK (2011). Differential protein expression in sugarcane during sugarcane-*Sporisorium scitamineum* interaction revealed by 2-DE and MALDI-TOF-TOF/MS. Com Func Genom.

[32] Wasternack C (2007). Jasmonates: an update on biosynthesis, signal transduction and action in plant stress response, growth and development. Ann Bot.

[33] Howe GA, Jander G (2008). Plant immunity to insect herbivores. Annu Rev Plant Biol.

[34] Browse J (2008). Jasmonate passes muster: a receptor and targets for the defense hormone. Annu Rev Plant Biol.

[35] Wasternack C, Kombrink E (2009). Jasmonates: structural requirements for lipid-derived signals active in plant stress responses and development. FEBS Lett.

[36] Devoto A, Turner JG (2003). Regulation of jasmonate-mediated plant responses in *Arabidopsis*. Ann Bot.

[37] Ishiga Y, Ishiga T, Uppalapati SR, Mysore KS (2013). Jasmonate ZIM-domain (JAZ) protein regulates host and nonhost pathogen-induced cell death in tomato and *Nicotiana benthamiana*. PLoS One.

[38] Berger S, Bell E, Mullet JE (1996). Two methyl jasmonate-insensitive mutants show altered expression of *AtVsp* in response to methyl jasmonate and wounding. Plant Physiol.

[39] Xie DX, Feys BF, James S, Nietorostro M, Turner JG (1998). COI1: an *Arabidopsis* gene required for jasmonate-regulated defense and fertility. Science.

[40] Staswick PE, Tiryaki I (2004). The oxylipin signal jasmonic acid is activated by an enzyme that conjugates it to isoleucine in *Arabidopsis*. Plant Cell.

[41] Xu LH, Liu FQ, Lechner E, Genschik P, Crosby W, Ma H (2002). The SCF(COI1) ubiquitin-ligase complexes are required for jasmonate response in *Arabidopsis*. Plant Cell.

[42] Thines B, Katsir L, Melotto M, Niu Y, Mandaokar A, Liu G (2007). JAZ repressor proteins are targets of the SCF(COI1) complex during jasmonate signalling. Nature.

[43] Chini A, Fonseca S, Fernández G, Adie B, Chico JM, Lorenzo O (2007). The JAZ family of repressors is the missing link in jasmonate signalling. Nature.

[44] Yan Y, Stolz S, Chételat A, Reymond P, Pagni M, Dubugnon L (2007). A downstream mediator in the growth repression limb of the jasmonate pathway. Plant Cell.

[45] Melotto M, Mecey C, Niu Y, Chung HS, Katsir L, Yao J (2008). A critical role of two positively charged amino acids in the Jas motif of *Arabidopsis* JAZ proteins in mediating coronatine- and jasmonoyl isoleucine-dependent interactions with the COI1 F-box protein. Plant J.

[46] Chini A. The ZIM domain mediates homo- and heteromeric interactions between *Arabidopsis* JAZ proteins. Plant J 2009;59:77-87. 10.1111/j.1365-313X.2009.03852.x 19309455

[47] Amanda Wager JB (2011). Social network: JAZ protein interactions expand ourknowledge of jasmonate signaling. Front Plant Sci.

[48] Kazan K, Manners JM (2012). JAZ repressors and the orchestration of phytohormone crosstalk. Trends Plant Sci.

[49] Bai YH, Meng YJ, Huang DL, Qi YH, Chen M (2011). Origin and evolutionary analysis of the plant-specific TIFY transcription factor family. Genomics.

[50] Vanholme B, Grunewald W, Bateman A, Kohchi T, Gheysen G (2007). The tify family previously known as ZIM. Trends Plant Sci.

[51] Katsir L, Schilmiller AL, Staswick PE, He SY, Howe GA (2008). From the cover: COI1 is a critical component of a receptor for jasmonate and the bacterial virulence factor coronatine. P Nat Acad Sci e USA.

[52] Demianski AJ, Chung KM, Kunkel BN (2012). Analysis of *Arabidopsis JAZ* gene expression during *Pseudomonas syringae* pathogenesis. Mol Plant Pathol.

[53] Chung HS, Koo AJ, Gao X, Jayanty S, Thines B, Jones AD (2008). Regulation and function of *Arabidopsis* Jasmonate ZIM-domain genes in response to wounding and herbivory. Plant Physiol.

[54] Ye HY, Du H, Tang N, Li XH, Xiong LZ (2009). Identification and expression profiling analysis of *TIFY* family genes involved in stress and phytohormone responses in rice. Plant Mol Biol.

[55] Zhu D, Cai H, Luo X, Bai X, Deyholos MK, Chen Q (2012). Over-expression of a novel *JAZ* family gene from *Glycine soja*, increases salt and alkali stress tolerance. Biochem Bioph Res Co..

[56] Oh Y, Baldwin IT, Gális I (2012). NaJAZh regulates a subset of defense responses against herbivores and spontaneous leaf necrosis in *Nicotiana attenuata* plants. Plant Physiol.

[57] Oh Y, Baldwin IT, Galis I (2013). A jasmonate ZIM-domain protein NaJAZd regulates floral jasmonic acid levels and counteracts flower abscission in *Nicotiana attenuata* plants. PLoS One.

[58] Dewey RE, Xie JH (2013). Molecular genetics of alkaloid biosynthesis in *Nicotiana tabacum*. Phytochemistry.

[59] Zhou XJ, Yan SW, Sun C, Li SZ, Li J, Xu MY (2015). A maize jasmonate Zim-domain protein, ZmJAZ14, associates with the JA, ABA, and GA signaling pathways in transgenic *Arabidopsis*. PLoS One.

[60] Zhang YC, Gao M, Singer SD, Fei ZJ, Wang H, Wang XP (2012). Genome-wide identification and analysis of the *TIFY* gene family in grape. PLoS One.

[61] Que YX, Su YC, Guo JL, Wu QB, Xu LP (2014). A global view of transcriptome dynamics during *Sporisorium scitamineum* challenge in sugarcane by RNA-seq. PLoS One.

[62] Moosawi-Jorf SA, Izadi MB (2007). In vitro detection of yeast-like and mycelial colonies of *Ustilago scitaminea* in tissue-cultured plantlets of sugarcane using polymerase chain reaction. J Appl Sci.

[63] Tamura K, Peterson D, Peterson N, Stecher G, Nei M, Kumar S (2011). MEGA5: molecular evolutionary genetics analysis using maximum likelihood, evolutionary distance, and maximum parsimony methods. Mol Biol & Evol.

[64] Que YX, Xu LP, Xu JS, Zhang JS, Zhang MQ (2009). Selection of control genes in real-time qPCR analysis of gene expression in sugarcane. Chin J Trop Crop.

[65] Ling H, Wu QB, Guo JL, Xu LP, Que YX (2014). Comprehensive selection of reference genes for gene expression normalization in sugarcane by real time quantitative RT-PCR. PLoS One.

[66] Livak KJ, Schmittgen TD (2001). Analysis of relative gene expression data using real-time quantitative PCR and the 2^-∆∆CT^ method. Methods.

[67] Su YC, Xu LP, Fu ZW, Yang YT, Guo JL, Wang SS (2014). *ScChi*, encoding an acidic class III chitinase of sugarcane, confers positive responses to biotic and abiotic stresses in sugarcane. Int J Mol Sci.

[68] Guo JL, Xu LP, Fang JP, Su YC, Fu HY, Que YX (2012). A novel dirigent protein gene with highly stem-specific expression from sugarcane, response to drought, salt and oxidative stresses. Plant Cell Rep.

[69] Farmer EE, Alméras E, Krishnamurthy V (2003). Jasmonates and related oxylipins in plant responses to pathogenesis and herbivory. Curr Opin Plant Biol.

[70] Rojo E, Solano R, Sánchez-Serrano JJ (2003). Interactions between signaling compounds involved in plant defense. J Plant Growth Regul.

[71] Qi TC, Song SS, Ren QC, Wu DW, Huang H, Chen Y (2011). The jasmonate-ZIM-domain proteins interact with the WD-repeat/bHLH/MYB complexes to regulate jasmonate-mediated anthocyanin accumulation and trichome initiation in *Arabidopsis thaliana*. Plant Cell.

[72] Chung HS, Niu Y, Browse J, Howe GA (2009). Top hits in hontemporary JAZ: an update on jasmonate signaling. Phytochemistry.

[73] Staswick PE (2008). JAZing up jasmonate signaling. Trends Plant Sci.

[74] Gimenez-Ibanez S, Boter M, Fernández-Barbero G, Chini A, Rathjen JP, Solano R. The bacterial effector hopX1 targets JAZ transcriptional repressors to activate jasmonate signaling and promote infection in *Arabidopsis*. PLoS Biol. 2014;12:2798–798. 10.1371/journal.pbio.1001792 PMC3928049 24558350

[75] Hong H, Xiao H, Yuan H, Zhai J, Huang X (2015). Cloning and characterisation of *JAZ* gene family in *Hevea brasiliensis*. Plant Biol.

[76] Bari R, Jones JDG (2009). Role of plant hormones in plant defence responses. Plant Mol Biol.

[77] Pieterse MJ, Leon-Reyes A, Ent SVD, Wees SCMV (2009). Networking by small-molecule hormones in plant immunity. Nat Chem Biol.

[78] Liu B, Xue XD, Cui SP, Zhang XY, Han QM, Zhu L (2009). Cloning and characterization of a wheat b-1,3-glucanase gene induced by the stripe rust pathogen *Puccinia striiformis* f. Sp. *tritici*. Mol Biol Rep.

[79] Katsir L, Chung HS, Koo AJ, Howe GA (2008). Jasmonate signaling: a conserved mechanism of hormone sensing. Curr Opin Plant Biol.

[80] Prat S, Willmitzer L. Abscisic acid: physiology and biochemistry. In: Davies WJ, Jones HG, editors. Oxford: BIOS Scientific Publishers; 1991. p. 201–16.

[81] Engelberth J, Contreras CF, Viswanathan S (2012). Transcriptional analysis of distant signaling induced by insect elicitors and mechanical wounding in *Zea mays*. PLoS One.

[82] Zhu D, Bai X, Chen C, Chen Q, Cai H, Li Y (2011). *GsTIFY10*, a novel positive regulator of plant tolerance to bicarbonate stress and a repressor of jasmonate signaling. Plant Mol Biol.

[83] Chaurasia N, Mishra Y, Rai LC (2008). Cloning expression and analysis of phytochelatin synthase (*pcs*) gene from *Anabaena* sp. PCC 7120 offering multiple stress tolerance in *Escherichia coli*. Biochem Bioph Res Co.

[84] Du SC, Hwang IS, Hwang BK (2012). Requirement of the cytosolic interaction between pathogenesis-related protein10 and leucine-rich repeat protein1 for cell death and defense signaling in pepper. Plant Cell.

[85] Melech-Bonfil S, Sessa G (2010). Tomato MAPKKKε is a positive regulator of cell-death signaling networks associated with plant immunity. Plant J.

[86] Li YZ, Tessaro MJ, Li X, Zhang YL (2010). Regulation of the expression of plant resistance gene *SNC1* by a protein with a conserved BAT2 domain. Plant Physiol.

[87] Levine A, Tenhaken R, Dixon R, Lamb C (1994). H_2_O_2_ from the oxidative burst orchestrates the plant hypersensitive disease resistance response. Cell.

[88] Ron M, Avni A (2004). The receptor for the fungal elicitor ethylene-inducing xylanase is a member of a resistance-like gene family in tomato. Plant Cell.

