# Supplementary figures and images for: Plant jasmonate ZIM domain genes: shedding light on structure and expression patterns of JAZ gene family in sugarcane
Source: BMC Genomics. 2017 Oct 11;18:771. doi: 10.1186/s12864-017-4142-3 (PMC5637078; doi:10.1186/s12864-017-4142-3)

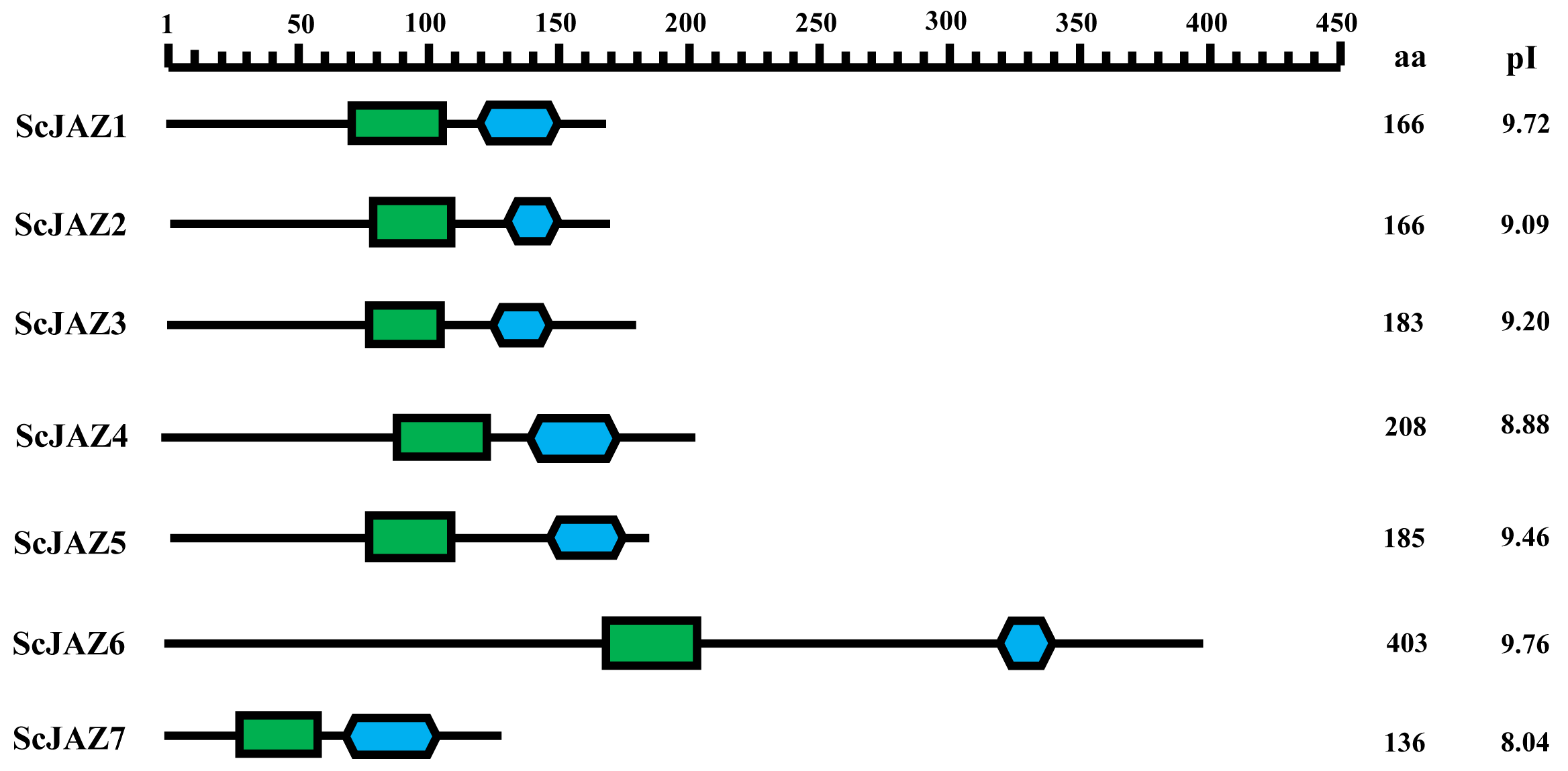

Supplement: Supplementary file 1 — The domain structure of the corresponding ScJAZ proteins. The conserved motifs of TIFY (TIF[F/Y]XG) and Jas (SLX2FX2KRX2RX5PY) presented among the seven sugarcane ScJAZ proteins. Green box: TIFY domain; Blue box: CCT_2 domain. aa: the number of amino acids; pI: isoelectric point. (TIFF 343 kb) [file 12864_2017_4142_MOESM1_ESM.tif]

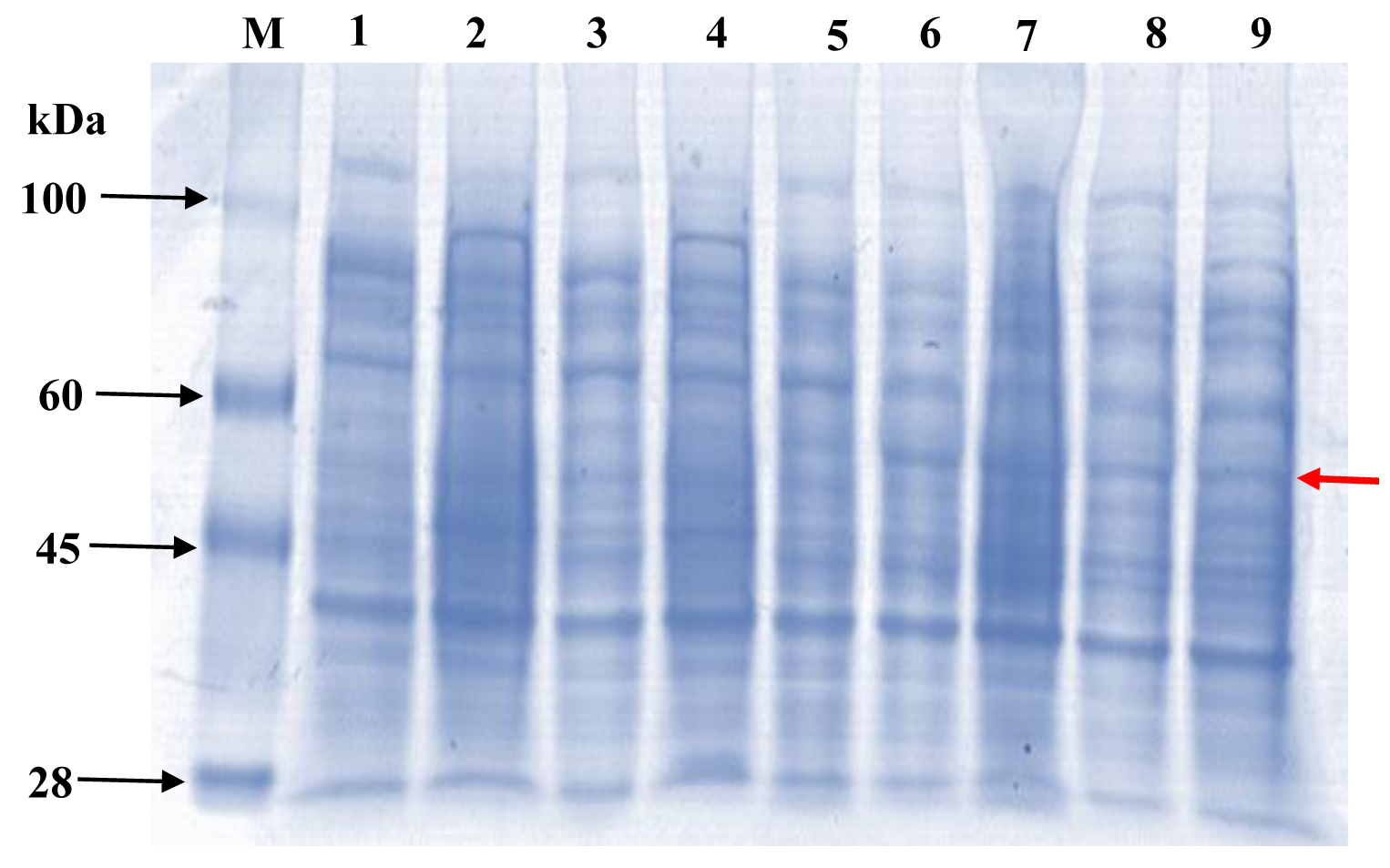

Supplement: Supplementary file 2 — The prokaryotic expression of pET 32a–ScJAZ6 fusion protein in Escherichia coli BL21 (DE3). M, protein marker; 1, blank (E. coli BL21 cells) without induction; 2, blank induction for 8 h; 3, control (BL21 pET 32a) without induction; 4, control induction for 8 h; 5, BL21 pET 32a–ScJAZ6 without induction; 6–9, BL21 pET 32a–ScJAZ6 induction for 2 h, 4 h, 6 h, and 8 h, respectively. The induced protein is indicated by a red arrow. (TIFF 3905 kb) [file 12864_2017_4142_MOESM2_ESM.tif]
